# Supplementary material for: Long-wave infrared transparent sulfur polymers enabled by symmetric thiol cross-linker
Source: Nat Commun. 2023 May 19;14:2866. doi: 10.1038/s41467-023-38398-5 (PMC10199000; doi:10.1038/s41467-023-38398-5)
Supplement: Supplementary file 1 — Supplementary Information [file 41467_2023_38398_MOESM1_ESM.pdf]

Supplementary Information

Long-wave infrared transparent sulfur polymers enabled by symmetric thiol cross-linker

Miyeon Lee[1,5], Yuna Oh[2], Jaesang Yu[2], Se Gyu Jang[3], Hyeonuk Yeo[4], Jong-Jin Park[5], Nam-Ho You[1,]*

[1]Carbon Composite Materials Research Center, Korea Institute of Science and Technology (KIST), Wanju 55324, Republic of Korea

[2]Institute of Advanced Composite Materials Research Center, Korea Institute of Science and Technology (KIST), Wanju 55324, Republic of Korea

[3]Functional Composite Materials Research Center, Korea Institute of Science and Technology (KIST), Wanju 55324, Republic of Korea

[4]Department of Chemistry Education, Kyungpook National University, Daegu 41566, Republic of Korea

[5]Department of Polymer Engineering, Chonnam National University, Gwangju 61186, Republic of Korea

Table of Contents

I) Supplementary Methods

A) Materials

B) Instruments for characterization

1. Thermal and mechanical properties
2. Chemical properties
3. Optical properties

C) Experimental procedures

1. Synthesis of a poly(S_{70}-*r*-BDT_{30}) copolymer
2. General procedure for synthesis of poly(S-*r*-BTT) copolymers
3. General procedure for synthesis of a poly(S-*r*-DIB) copolymers
4. General procedure for synthesis of a poly(S-*r*-DVB) copolymers
5. General procedure for the preparation of bulk polymer free-standing thin films
6. General procedure for the preparation of 1 mm thick polymer windows (Dia. 25 mm × Thickness 1 mm)

II) Supplementary Data

A) Properties of poly(S_{70}-*r*-BDT_{30}) copolymer

B) Properties of poly(S_{90}-*r*-BTT_{10}) copolymer

C) Solid state NMR of poly(S-*r*-BTT) copolymers

D) Glass transition temperature (T_g) of poly(S_{70}-*r*-DIB_{30}), poly(S_{70}-*r*-DVB_{30}) and poly(S_{70}-*r*-BTT_{30}) copolymers

E) Solubility test of poly(S_{70}-*r*-DIB_{30}), poly(S_{70}-*r*-DVB_{30}) and poly(S_{70}-*r*-BTT_{30}) copolymers

F) MD simulations of poly(S-*r*-BTT) copolymers

G) DFT calculation of models compounds

H) DMA curves of poly(S-*r*-BTT) thin films

I) Refractive indices of poly(S-*r*-BTT) and poly(S-*r*-DVB) thin films

J) FT-IR transmission (%) spectra of Ge, commercial polymers and S70-DIB30 1 mm thick windows

K) FT-IR transmission (%) spectra of S-DVB and S-BTT 1 mm thick windows

L) Average FT-IR transmission (%) spectrum of Ge and polymer windows

M) FT-IR transmission (%) spectra of S70-BTT30 windows according to hot-pressing condition

N) Optical stability of S70-DIB30, S70-DVB30 and S70-BTT30 windows

O) Elemental analyses of poly(S_{70}-*r*-DIB_{30}), poly(S-*r*-DVB) and poly(S-*r*-BTT) copolymers

P) Infrared (IR) imaging experiments

III) Supplementary References

I) Supplementary Methods

A) Materials

Sulfur (S_8, powder, 98.0 % extra pure, SAMCHUN), 1,3,5-Benzenetrithiol (BTT, >98.0 % (GC), TCI), 1,3-Benzenedithiol (BDT, >95.0 % (GC), TCI), 1,3-Diisopropenylbenzene (DIB, >97.0 % (GC), TCI), Divinylbenzene (DVB, 80.0 % technical grade, Aldrich), Polyethylene (PE, low density, Aldrich), Poly(methyl methacrylate) (PMMA, Aldrich), Cyclic olefin polymer (ARTON D4000, JSR Corporation), Polyimide film (Kapton), Dichloromethane (DCM) and Tetrahydrofuran (THF) were purchased and used as received. Uncoated Germanium windows (Ge, Dia. 25.00 (mm) × Thickness 1.00 (mm)) and USAF 1951 target were purchased from Edmund Optics. Various patterned PMMA masks were prepared as target for LWIR imaging.[1]

B) Instruments for characterization

1. Thermal and mechanical properties

Differential scanning calorimetry (DSC, DSC 8000, PerkinElmer, U.S.A) was carried out under N_2 flow at a heating (cooling) rate of 10 °C/min from -45 °C to 165 °C. Thermogravimetric analysis (TGA, Setaram Solutions, France) was performed under N_2 flow at a heating rate of 10 °C /min from 30 °C to 600 °C. Dynamic mechanical analysis (DMA, DMA Q800, TA Instruments, U.S.A) was carried out -45 °C to 165 °C at a scanning rate of 5 °C/min with a preload of 0.01 N. The specimens were prepared thin film (30 mm length, 10 mm wide, and ca. 0.4 mm thickness).

2. Chemical properties

Solid-state ^{13}C NMR CP/MAS spectra were measured using solid 500 MHz NMR (Bruker Avance III HD, Bruker, German) with 4 mm CPMAS probes. Spinning at 5 kHz and pulse repetition delays of 5 s were performed. Elemental analyzer (EA, IT/Flash 2000, Thermo Fisher Scientific, U.S.A) was conducted to determine the C, H, N, S content of the copolymers synthesized by inverse vulcanization. X-ray diffractometer (XRD, SmartLab, Rigaku Corporation, Japan) was performed in the 2Theta/Theta mode from 5 ° to 80 ° (200 mA, 45 kV). Attenuated total reflectace–Fourier transforminfrared (ATR-FTIR) spectra were obtained with a Nicolet iS10 (Thermo Fisher Scientific, U.S.A) with 32 scans per spectrum from 4000-550 cm^{-1}.

3. Optical properties

Transmittance–Fourier transforminfrared (Transmittance-FTIR) spectra of polymer windows were recorded with a Spectrum 100 (PerkinElmer, U.S.A) with 16 scans per spectrum from 4000-400 cm^{-1}, or Nicolet iS10 (Thermo Fisher Scientific, U.S.A) with 72 scans per spectrum from 4000-500 cm^{-1}. IR transmission (%) of Mid-wave infrared (MWIR) region (3–5 μm, 3300-2000 cm^{-1}) and Long-wave infrared (LWIR) region (7–14 μm, 1420-710 cm^{-1}) were calculated from average Y value of each spectral region. Refractive indices of polymer films were measured using a prism coupler (PC-2000, Metricon Corporation, U.S.A). The In-plane/out-of-plane birefringence calculated as $\Delta n = n_{TE} - n_{TM}$ and the average refractive index (n_{av}) was calculated using the following equation. $n_{av} = [(2n_{TE}^2 + n_{TM}^2)/^3]^{1/2}$. The Abbe's number is given by as $V_{NIR} = (n_{829} - 1)/(n_{637} - n_{1306})$. X-ray microscopy (XRM, Zeiss Xradia 520 Versa, Germany) with the parameters of 40.21 kV voltage and 3.03 W power was used to image the internal features of the polymer window. IR imaging experiments were performed using various objects (e. g. female subject, USAF 1951 target and various pattered PMMA mask). Near infrared (NIR) imaging was performed using a Digital Night Vision Binocular (APL-NV001+, Apexel Technology, China) in IR illumination of 850 nm. Mid-wave infrared (MWIR) images were captured using a Themos mini(C-10614-02) thermal emission microscope (Hamamatsu Photonics, Japan) with a 3−5 μm lens. Long-wave infrared (LWIR) images were taken using a FLIR T335 (Teledyne FLIR, U.S.A) with a 7.5−13 μm wavelength range.

C) Experimental procedures

1. Synthesis of a poly(S_{70}-*r*-BDT_{30}) copolymer

To a glass vial equipped with a magnetic stir bar was added sulfur (S_8, 2.8 g, 70 wt%) and heated in a 185 °C oil bath until yellow sulfur powder turn into orange liquid sulfur. 1,3-Benzenedithiol (BDT, 1.2 g, 0.97 ml, 30 wt%) was added directly to the liquid sulfur via syringe. The sulfur and BDT mixture reacted in a 185 °C oil bath, generating vigorous gas. After 1 hour, the glass vial with product was quenched in liquid nitrogen bath and product was separated from the vial. The product is obtained as a yellow solid.

2. General procedure for synthesis of poly(S-*r*-BTT) copolymers

To a glass vial equipped with a magnetic stir bar was added sulfur (S_8) and heated in a 185 °C oil bath until yellow sulfur powder turn into orange liquid sulfur. 1,3,5-Benzenetrithiol (BTT) was added directly to the liquid sulfur. The sulfur and BTT mixture reacted in a 185 °C oil bath, generating vigorous gas. After 1 hour, the glass vial with product was quenched in liquid nitrogen bath and product was separated from the vial. All experimental procedures were carried out in a fume hoods. It should be noted that toxic H_2S gas generated during inverse vulcanization, requiring operator care for safety.

a. Synthesis of poly(S_{90}-*r*-BTT_{10}): The synthesis was carried out by following the general procedure written above with S_8 (3.6 g, 90 wt%) and BTT (0.4 g, 10 wt%) to obtain a yellow solid.

b. Synthesis of poly(S_{80}-*r*-BTT_{20}): The synthesis was carried out by following the general procedure written above with S_8 (3.2 g, 80 wt%) and BTT (0.8 g, 20 wt%) to obtain a yellow glassy solid.

c. Synthesis of poly(S_{70}-*r*-BTT_{30}): The synthesis was carried out by following the general procedure written above with S_8 (2.8 g, 70 wt%) and BTT (1.2 g, 30 wt%) to obtain a yellow glassy solid.

d. Synthesis of poly(S_{60}-*r*-BTT_{40}): The synthesis was carried out by following the general procedure written above with S_8 (2.4 g, 60wt%) and BTT (1.6 g, 40 wt%) to obtain a yellow glassy solid.

e. Synthesis of poly(S_{50}-*r*-BTT_{50}): The synthesis was carried out by following the general procedure written above with S_8 (2.0 g, 50 wt%) and BTT (2.0 g, 50 wt%) to obtain a yellow glassy solid.

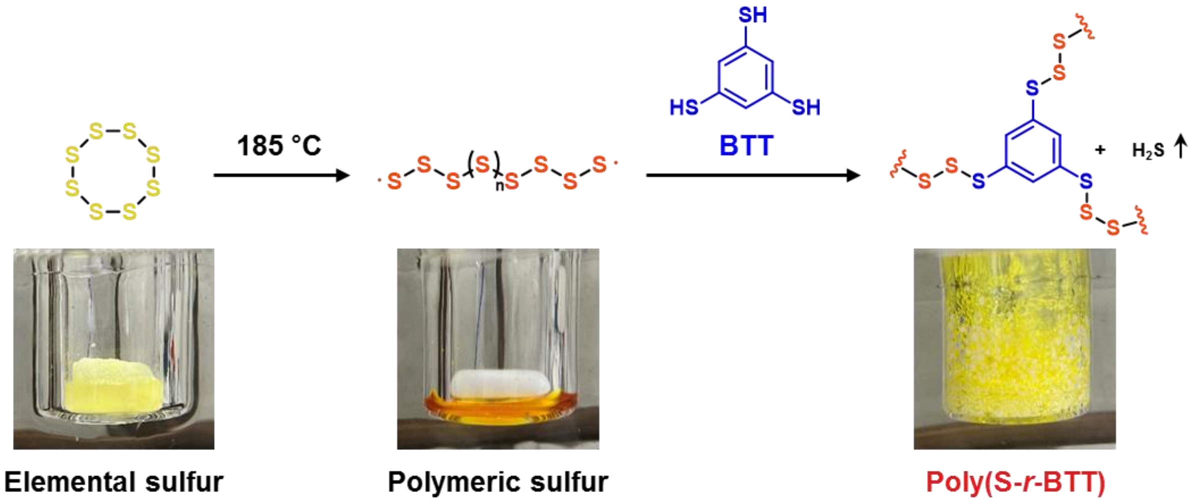

Supplementary figure 1. Synthesis of poly(S-*r*-BTT) copolymer via inverse vulcanization.

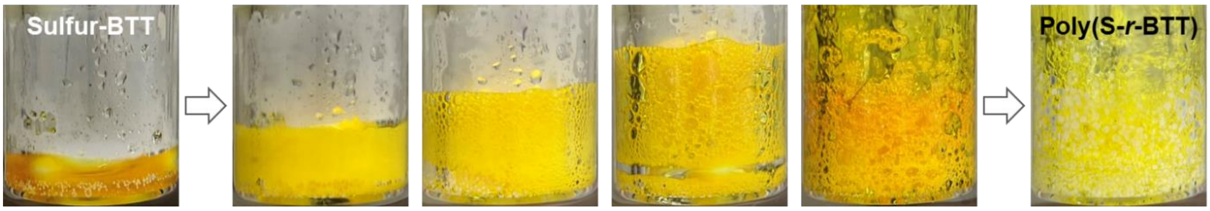

Supplementary figure 2. Digital image showing the process of S-BTT mixture curing into a poly(S-*r*-BTT) in a 185 °C oil bath.

3. General procedure for synthesis of a poly(S-*r*-DIB) copolymers

To a glass vial equipped with a magnetic stir bar was added sulfur (S_8) and heated in a 185 °C oil bath until yellow sulfur powder turn into orange liquid sulfur. 1,3-Diisopropenylbenzene (DIB) was added directly to the liquid sulfur via syringe. The sulfur and DIB mixture reacted in a 185 °C oil bath for 1 hour. The glass vial with product was quenched in liquid nitrogen bath and product was separated from the vial.

a. Synthesis of poly(S_{90}-*r*-DIB_{10}): The synthesis was carried out by following the general procedure written above with S_8 (3.6 g, 90 wt%) and DIB (0.4 g, 0.43 ml, 10 wt%) to obtain a cherry-colored solid.

b. Synthesis of poly(S_{85}-*r*-DIB_{15}): The synthesis was carried out by following the general procedure written above with S_8 (3.4 g, 85 wt%) and DIB (0.6 g, 0.65 ml, 15 wt%) to obtain a cherry-colored solid.

c. Synthesis of poly(S_{80}-*r*-DIB_{20}): The synthesis was carried out by following the general procedure written above with S_8 (3.2 g, 80 wt%) and DIB (0.8 g, 0.87 ml, 20 wt%) to obtain a cherry-colored solid.

d. Synthesis of poly(S_{70}-*r*-DIB_{30}): The synthesis was carried out by following the general procedure written above with S_8 (2.8 g, 70 wt%) and DIB (1.2 g, 1.30 ml, 30 wt%) to obtain a cherry-colored solid.

4. General procedure for synthesis of a poly(S-*r*-DVB) copolymers

To a glass vial equipped with a magnetic stir bar was added sulfur (S_8) and heated in a 160 °C oil bath until yellow sulfur powder turn into orange liquid sulfur. Divinylbenzene (DVB) was added directly to the liquid sulfur via syringe. The sulfur and DIB mixture reacted in a 160 °C oil bath for 1 hour. The glass vial with product was quenched in liquid nitrogen bath and product was separated from the vial.

a. Synthesis of poly(S_{90}-r-DVB_{10}): The synthesis was carried out by following the general procedure written above with S_8 (3.6 g, 90 wt%) and DVB (0.4 g, 0.44 ml, 10 wt%) to obtain a brown solid.

b. Synthesis of poly(S_{85}-*r*-DVB_{15}): The synthesis was carried out by following the general procedure written above with S_8 (3.4 g, 85 wt%) and DVB (0.6 g, 0.66 ml, 15 wt%) to obtain

a brown solid.

c. Synthesis of poly(S_{80}-*r*-DVB_{20}): The synthesis was carried out by following the general procedure written above with S_8 (3.2 g, 80 wt%) and DVB (0.8 g, 0.88 ml, 20 wt%) to obtain a brown glassy solid.

d. Synthesis of poly(S_{70}-*r*-DVB_{30}): The synthesis was carried out by following the general procedure written above with S_8 (2.8 g, 70 wt%) and DVB (1.2 g, 1.32 ml, 30 wt%) to obtain a brown glassy solid.

5. General procedure for the preparation of bulk polymer into free-standing thin films

The bulk polymer samples prepared as written above and commercial polymers were hot-pressed between two polyimide films with a stainless steel plate under a pressure of 5 MPa for 10 minutes (specific hot-pressing temperatures noted below).

a. Processing of poly(S_{70}-*r*-BDT_{30}) into free-standing thin film: The S70-BDT30 thin film was prepared via the general procedure described above at hot-pressing temperature T = 30 °C.

b. Processing of poly(S-*r*-BTT) into free-standing thin films: The S-BTT thin films were prepared via the general procedure described above at hot-pressing temperature T = 185 °C.

c. Processing of poly(S-*r*-DIB) into free-standing thin films: The S-DIB thin films were prepared via the general procedure described above at hot-pressing temperature T = 50-85 °C.

d. Processing of poly(S-*r*-DVB) into free-standing thin films: The S-DVB thin films were prepared via the general procedure described above at hot-pressing temperature T = 150-160 °C.

e. Processing of commercial polymers and ARTON D4000 into free-standing thin films: The commercial polymers (i.e. Polyethylene (PE) and Poly(methyl methacrylate) (PMMA)) and ARTON D4000 thin film were prepared via the general procedure described above at hot-pressing temperature T = 180-200 °C.

6. General procedure for the preparation of 1 mm thick polymer windows (Dia. 25 mm × Thickness 1 mm)

The bulk polymer samples prepared as written above and commercial polymers were hot-pressed between two polyimide films with a stainless steel mold (Dia. 25 mm × Thickness 1 mm) under a pressure of 20 MPa for 30 minutes (specific hot-pressing temperatures noted below). After cooling to room temperature, the polymer window was carefully removed from the mold. The polymer window thickness was measured for 3-point using an ABS digital thickness gauge (MITUTOYO, Japan). See tables below for thickness specific to each polymer windows.

a. Processing for 1 mm thick windows of poly(S_{70}-*r*-BDT_{30}): The S70-BDT30 windows was prepared via the general procedure described above at hot-pressing temperature T = 30 °C.

b. Processing for 1 mm thick windows of poly(S-*r*-BTT): The S-BTT windows were prepared via the general procedure described above at hot-pressing temperature T = 185 °C.

c. Processing for 1 mm thick windows of poly(S-*r*-DIB): The S-DIB windows were prepared via the general procedure described above at hot-pressing temperature T = 50-85 °C.

d. Processing for 1 mm thick windows of poly(S-*r*-DVB): The S-DVB windows were prepared via the general procedure described above at hot-pressing temperature T = 150-160 °C.

e. Processing for 1 mm thick windows of commercial polymers and ARTON D4000: The commercial polymers (i.e. Polyethylene (PE) and Poly(methyl methacrylate) (PMMA)) and ARTON D4000 windows were prepared via the general procedure described above at hot-pressing temperature T = 180-200 °C.

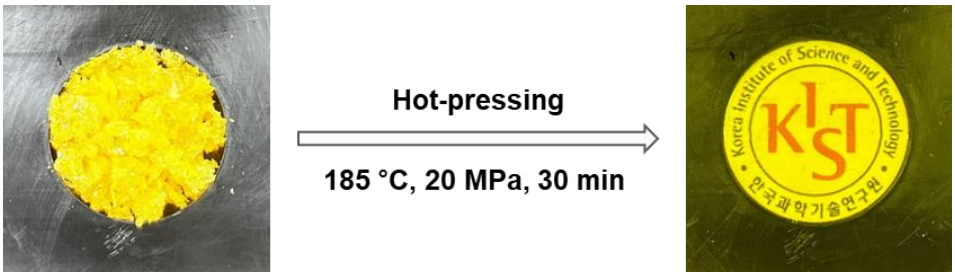

Supplementary figure 3. Photograph of the hot-pressed S70-BTT30 window using stainless steel mold (Dia. 25 mm × Thickness 1 mm)

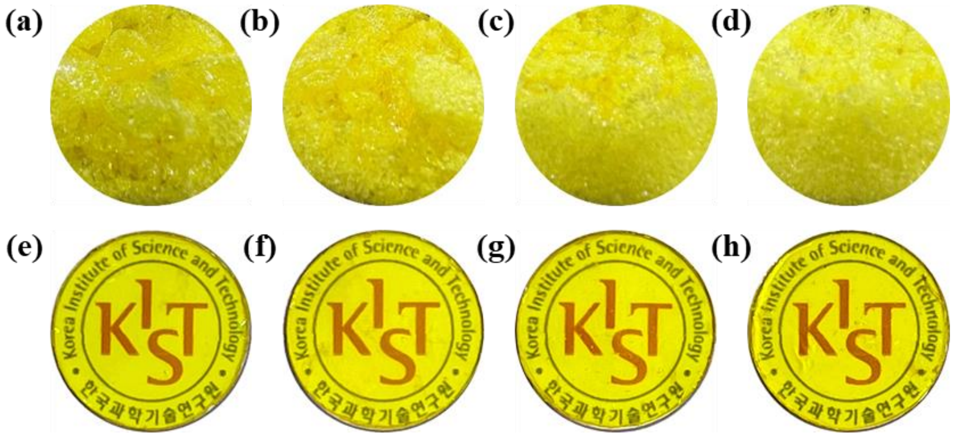

Supplementary figure 4. Photographs of bulk poly(S-*r*-BTT) and poly(S-*r*-BTT) windows (thickness ca. 1 mm) (a)&(e) S80-BTT20, (b)&(f) S70-BTT30, (c)&(g) S60-BTT40 and (d)&(h) S50-BTT50.

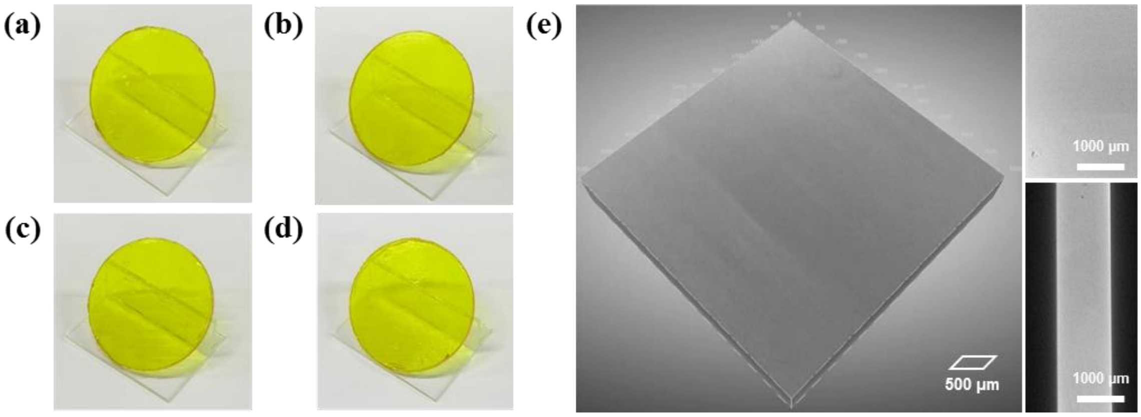

Supplementary figure 5. Photographs of poly(S-*r*-BTT) windows according to sulfur content (thickness ca. 1 mm) (a) S80-BTT20, (b) S70-BTT30, (c) S60-BTT40, (d) S50-BTT50 and (e) 3D X-ray microscope image of S70-BTT30 window.

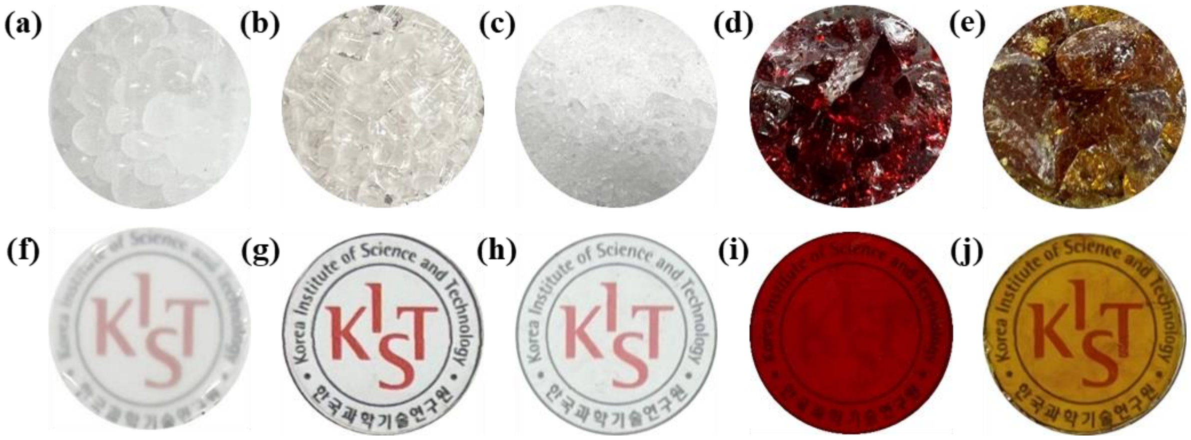

Supplementary figure 6. Photographs of bulk polymers and polymer windows (thickness ca. 1 mm) (a)&(f) PE (polyethylene), (b)&(g) COP (cyclic olefin copolymer), (c)&(h) PMMA (poly(methyl methacrylate)), (d)&(i) S70-DIB30 and (e)&(j) S70-DVB30.

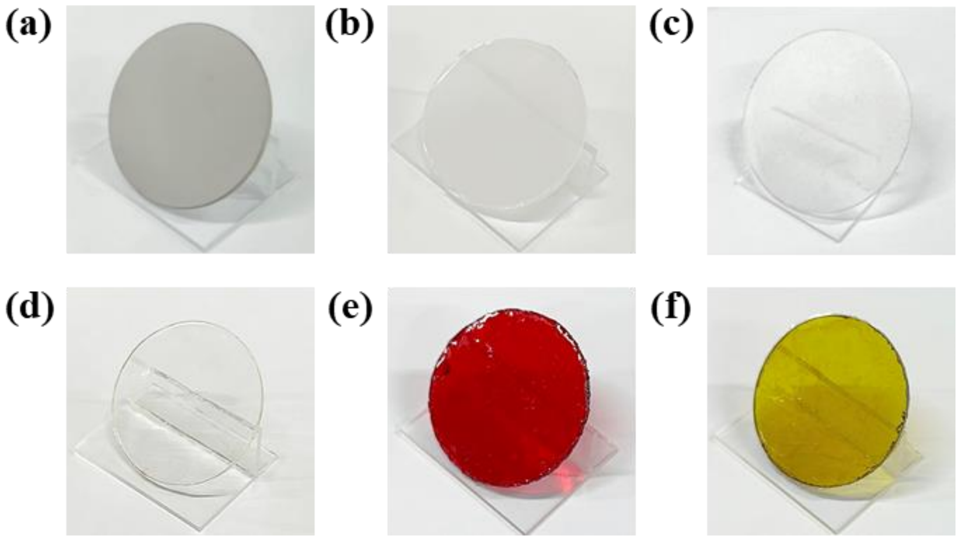

Supplementary figure 7. Photographs of Ge and polymer windows (thickness ca. 1 mm) (a) Ge window, (b) PE (polyethylene), (c) PMMA (poly(methyl methacrylate)), (d) COP (cyclic olefin copolymer), (e) S70-DIB30 and (f) S70-DVB30.

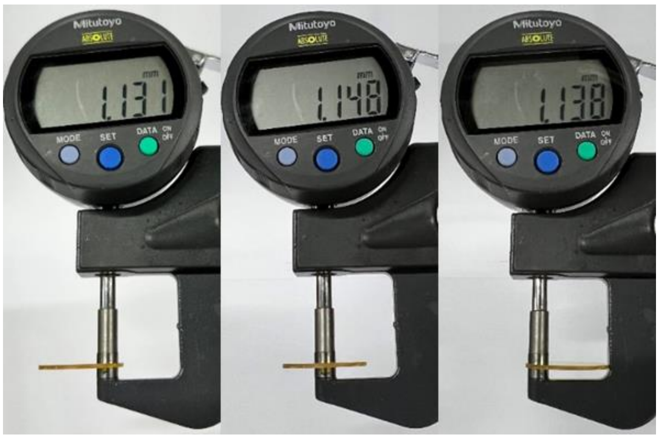

Supplementary figure 8. Digital image of S70-BTT30 window thickness measurement using an ABS Digital Thickness Gauge (MITUTOYO, Japan).

Supplementary table 1. Average thickness of the Ge window and polymer windows.

	**Thickness (mm)***
**Ge (Germanium)**	1.087
**PE (Polyethylene)**	1.090
**COP (Cyclic olefin copolymer)**	1.098
**PMMA (Poly(methyl methacrylate))**	1.153
**S70-DIB30**	1.281
**S90-DVB10**	1.083
**S85-DVB15**	1.148
**S80-DVB20**	1.266
**S70-DVB30**	1.276
**S80-BTT20**	1.156
**S70-BTT30**	1.139
**S60-BTT40**	1.144
**S50-BTT50**	1.156

* Average of 3-point measurements, using an ABS Digital Thickness Gauge (MITUTOYO, Japan).

For the purchased Ge window and the fabricated polymer windows, the thicknesses of the three points were measured as shown in figure S8, and the average values were summarized in Supplementary table 1. The poly(S-*r*-DVB) and poly(S-*r*-BTT) copolymers have been successfully prepared in window. However, the poly(S-*r*-DIB) copolymers has low glass transition temperature (T_g) and they were difficult to maintain the window shape in sulfur content of 90~70 wt% as is well known.[2] Therefore, the comparison of optical properties according to the structure of the cross-linker described below in the manuscript was centered on DVB and BTT.

II) Supplementary Data

A) Properties of poly(S_{70}-*r*-BDT_{30}) copolymer

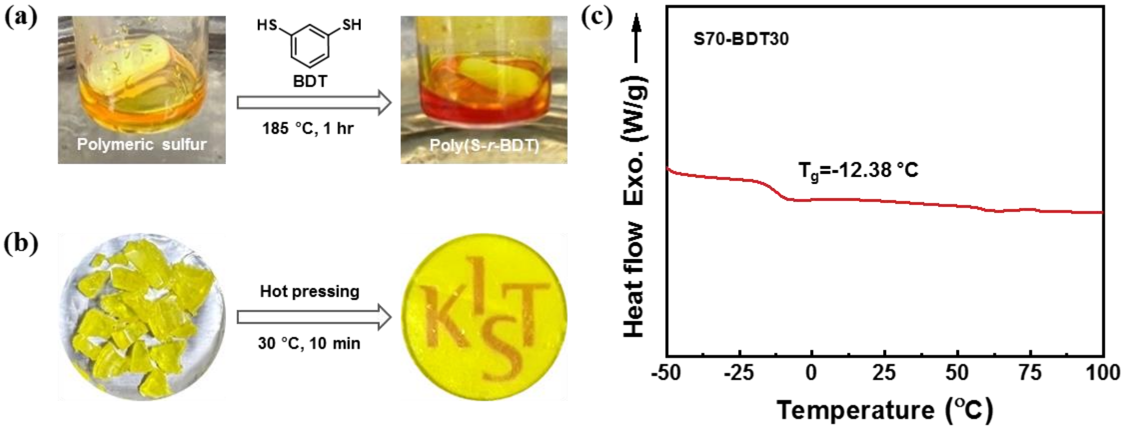

Supplementary figure 9. (a) Synthesis of poly(S_{70}-*r*-BDT_{30}) copolymer via inverse vulcanization, (b) photographs of hot-pressing of S70-BDT30 window (thickness: 1.187 mm) and (c) DSC curve indicating the glass transition temperatures (T_g) of S70-BDT30.

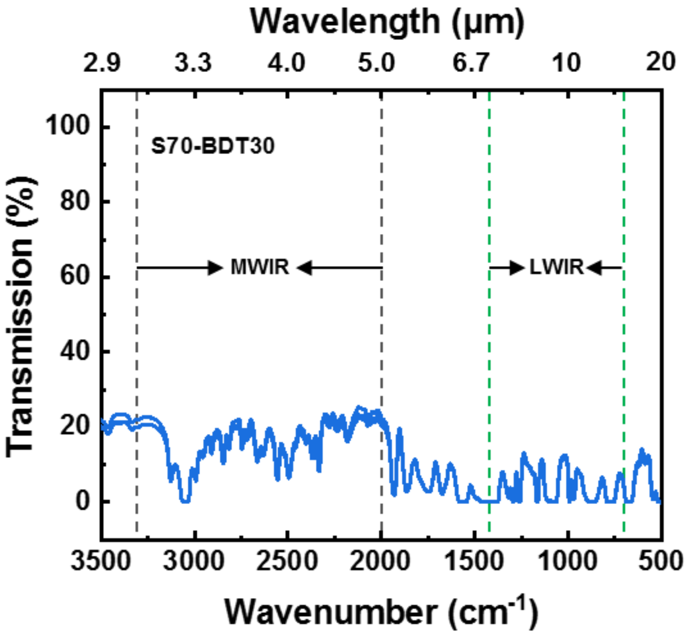

Supplementary figure 10. FT-IR transmission (%) spectra of the S70-BDT30 windows (3-point measurements).

Supplementary table 2. Optical properties of the poly(S-*r*-BDT) windows.

	**Thickness (mm)**	**Refractive index (n_{TE})***
**S70-BDT30**	1.187	1.92911

*Measured at 637 nm

As described in the manuscript, we first confirmed the reactivity of sulfur and thiol monomer using 1,3-benzenedithiol (BDT). Elemental sulfur and BDT cross-linker were successfully inverse vulcanized via condensation reaction at 185 °C. About 1 mm thick S70-BDT30 (70 wt% sulfur and 30 wt% BDT) window was prepared by low-temperature pressing of the yellow bulk copolymer. The S70-BDT30 window had a low glass transition temperature (T_g), making it difficult to persist shape at room temperature, so detailed optical characterization was not performed (Supplementary figure 9). Nevertheless, a high refractive index of n_{TE}>1.92 at 637 nm and IR transparency were confirmed (Supplementary figure 10 and Supplementary table 2).

B) Properties of poly(S_{90}-*r*-BTT_{10}) copolymer

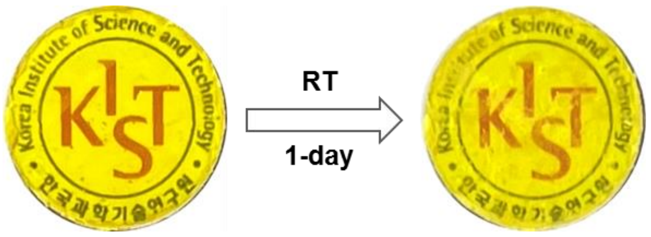

Supplementary figure 11. After 1-day storage at room temperature, the S90-BTT10 window observed crystallization due to unreacted sulfur (thickness ca. 1 mm).

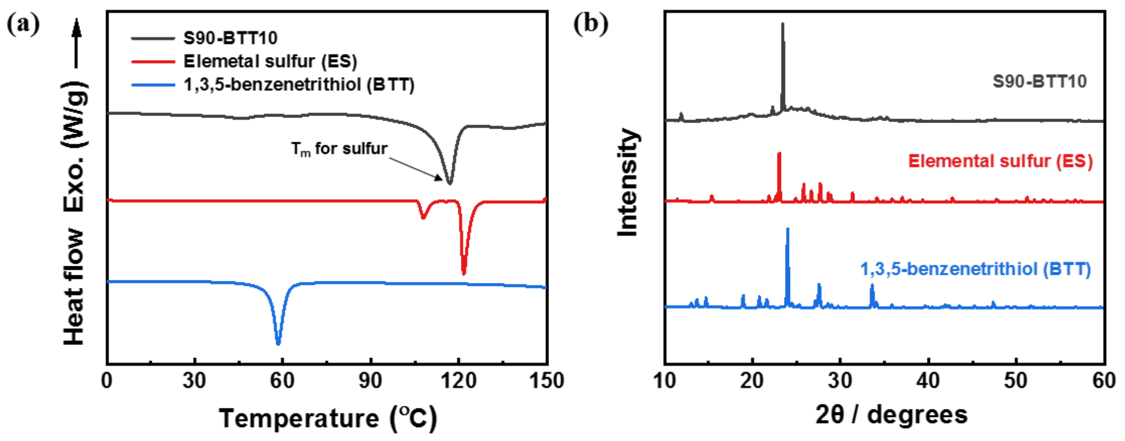

Supplementary figure 12. Thermal and chemical properties of the S90-BTT10 (a) DSC curves of 1st heating cycle and (b) XRD spectrum of S90-BTT10 (dark gray), elemental sulfur (red), 1,3,5-benzenetrithiol (blue).

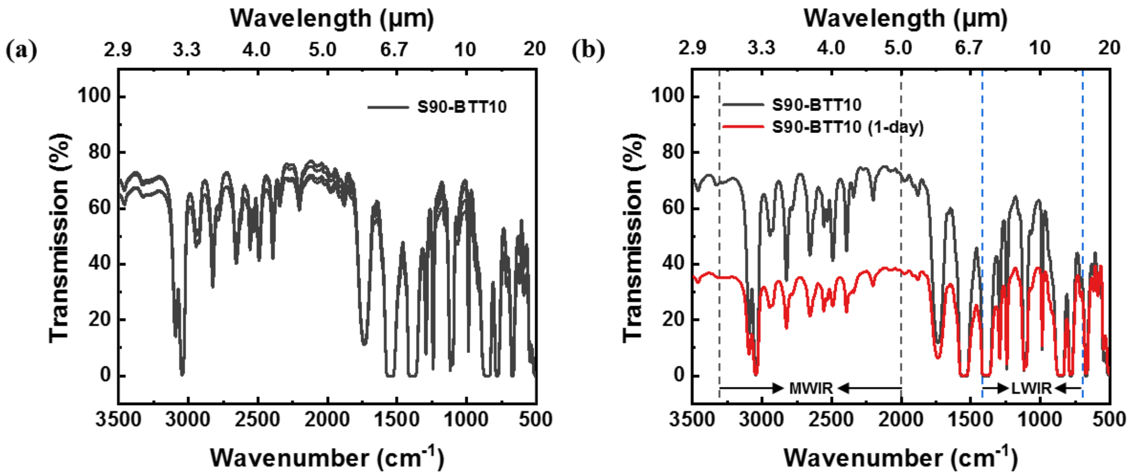

Supplementary figure 13. FT-IR transmission (%) spectra of the S90-BTT10 window (thickness: 1.100 mm) (a) initial S90-BTT10 window measured 5 times and (b) initial and 1-day room temperature storage S90-BTT10 window.

Crystallization of unreacted sulfur, which was not observed in sulfur content of 80~50 wt% copolymer, was clearly observed in the S90-BTT10 copolymer (Supplementary figures 11 and 12). The results summarized in Supplementary figure 13 showed the high MWIR and LWIR transmittance of the S90-BTT10 window. In contrast, S90-BTT10 windows stored for 1-day at room temperature showed significantly reduced IR transmittance due to the presence of residual unreacted sulfur (Supplementary figure 13).

C) Solid state NMR of poly(S-*r*-BTT) copolymers

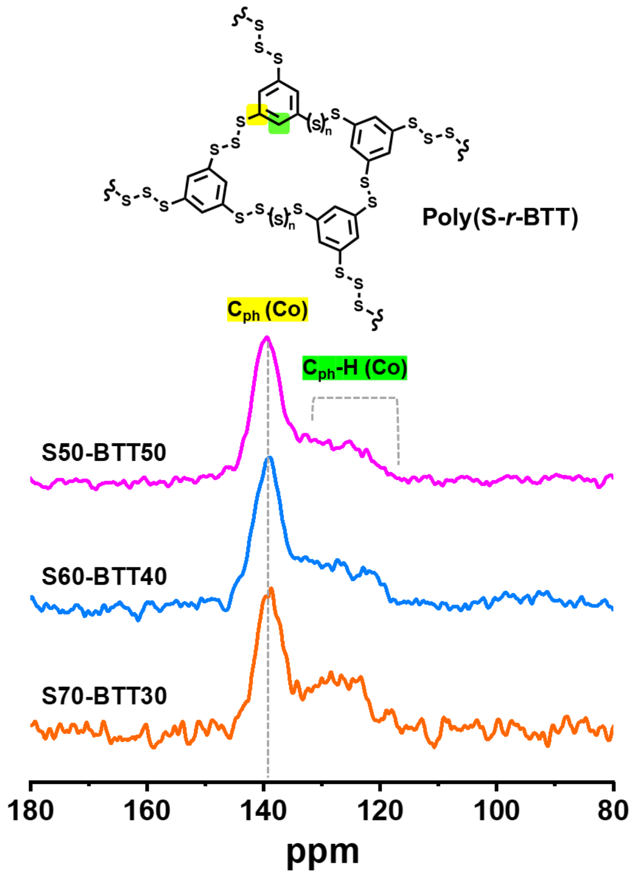

Supplementary figure 14. Solid-state ^{13}C NMR CP/MAS spectra of poly(S-*r*-BTT) copolymers.

The structure of poly(S-*r*-BTT) copolymers (70~50 wt% sulfur content) was proposed using a solid-state ^{13}C NMR CP/MAS spectra. Two signals corresponding to aromatic carbons were observed in the spectrum of the poly(S-*r*-BTT) copolymers. As shown in Supplementary figure 14, the peak at ca. 140 ppm and broad peaks at 120-130 ppm were assigned to C_{ph} (Co) and C_{ph}-H (Co), respectively. The peaks related to other organic moieties were not observed.

D) Glass transition temperature (T_g) of poly(S_{70}-*r*-DIB_{30}), poly(S_{70}-*r*-DVB_{30}) and poly(S_{70}-*r*-BTT_{30}) copolymers

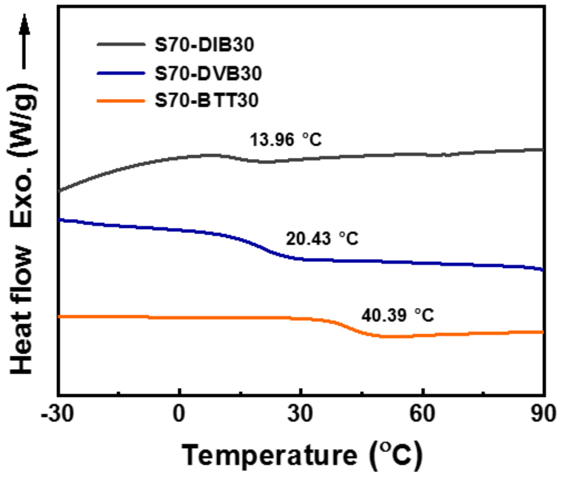

Supplementary figure 15. DSC curves of 2nd heating cycles indicating the glass transition temperatures (T_g) of S70-DIB30, S70-DVB30 and S70-BTT30.

E) Solubility test of poly(S_{70}-*r*-DIB_{30}), poly(S_{70}-*r*-DVB_{30}) and poly(S_{70}-*r*-BTT_{30}) copolymers

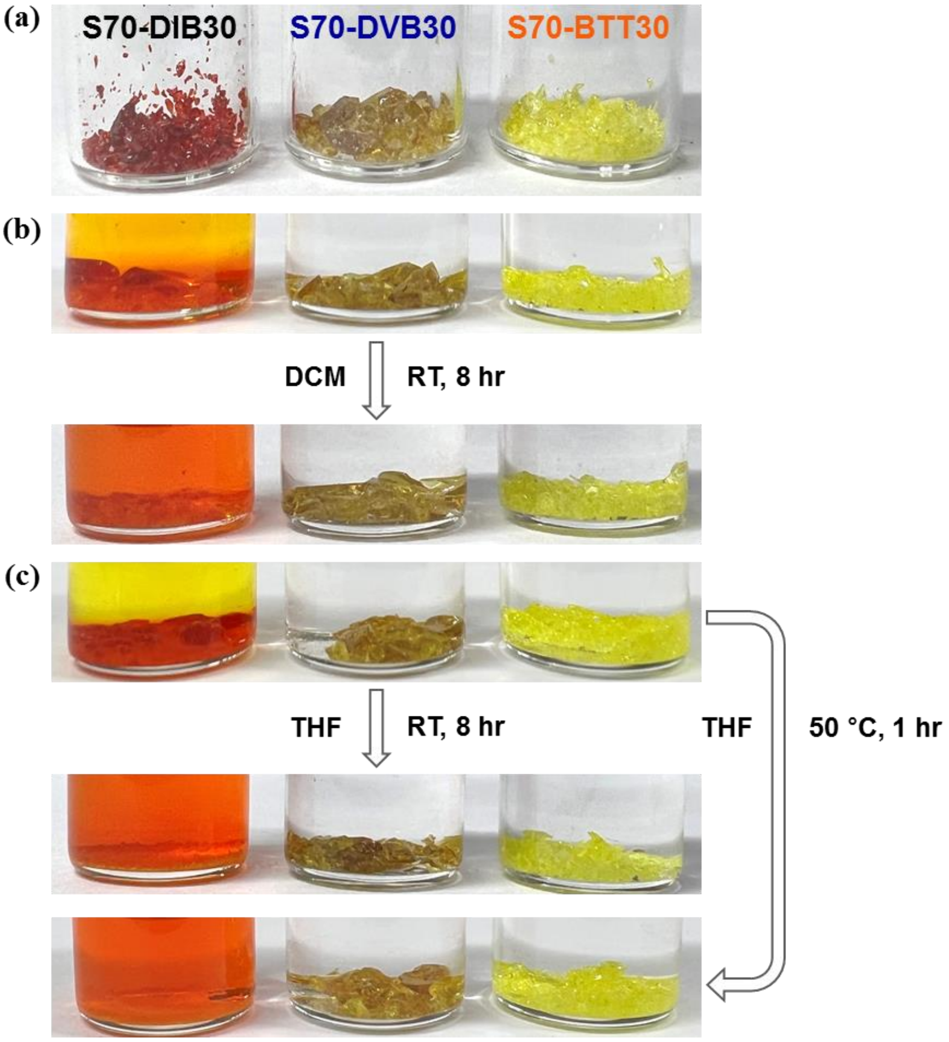

Supplementary figure 16. Solubility test of the S70-DIB30, S70-DVB30 and S70-BTT30 (from left to right) (a) bulk copolymers, (b) Dichloromethane (DCM) and (c) Tetrahydrofuran (THF).

F) MD simulations of poly(S-*r*-BTT) copolymer

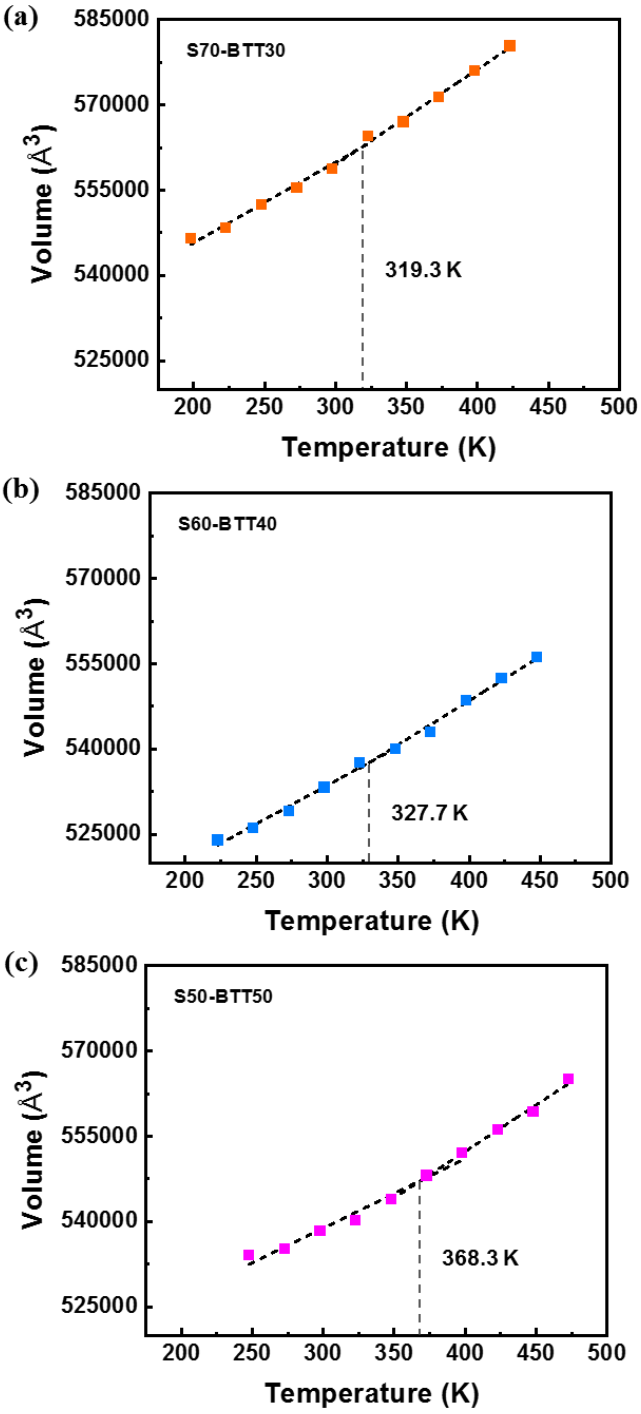

Supplementary figure 17. Glass transition temperatures of poly(S-*r*-BTT) copolymers calculated by the MD simulations (a) S70-BTT30, (b) S60-BTT40 and (c) S50-BTT50.

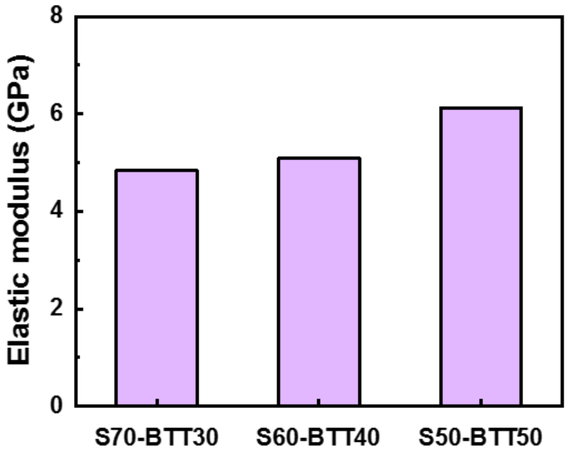

Supplementary figure 18. Elastic moduli of S-BTT models calculated by the MD simulations.

The elastic moduli of poly(S-*r*-BTT) copolymers were calculated by the MD simulations using the constant-strain method.[3] The elastic moduli of S-BTT models according to the contents of the sulfur and the BTT were 4.8, 5.1, and 6.1 GPa for the S70-BTT30, S60-BTT40, and S50-BTT50 models, respectively. The elastic modulus of the S50-BTT50 model increased up to 27 % compared to that of the S70-BTT30 model. This result related to the rigidity of the polymer chain. The rigidity of the polymer chain containing the sulfur increased by the addition of the BTT as the organic cross-linker. The increase of the rigidity of the flexible polymer chain led to the improvement of the elastic modulus of the poly(S-*r*-BTT) copolymer. Therefore, the S50-BTT50 model with the high BTT content has the highest elastic modulus.

G) DFT calculation of models compounds

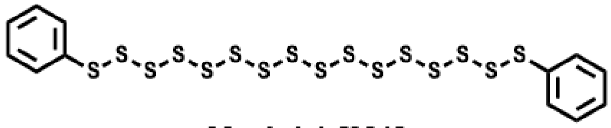

Model 1 [M1]

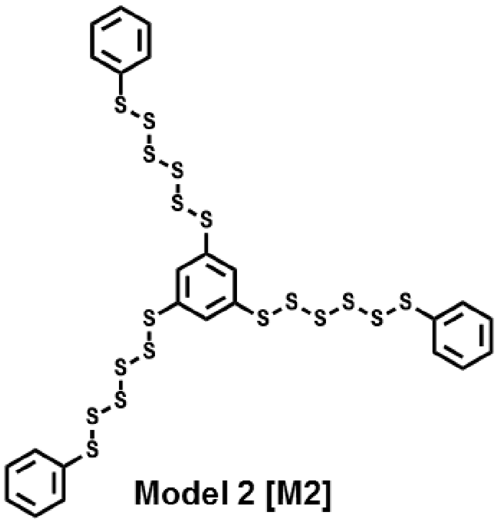

Model 2 [M2]

Supplementary figure 19. Chemical structures of model compounds, model 1 (M1) and model 2 (M2).

Supplementary table 3. DFT calculation results with model compounds on molecular volume, polarizability, and refractive index.

Sample	Molecular Volume (V_{mol})[a] [$Bohr^3$/mol]	Polarizability (α_λ)[b] [$Bohr^3$]				Refractive Index (n_λ)[c]					
		637 nm	829 nm	1306 nm	1549 nm	637 nm	829 nm	1306 nm	1549 nm	n_∞[d]	D[d] [×10^4]
**Model 1 [$PhS_{16}Ph$]**	4577.572	554.40	513.98	491.27	487.49	2.022	1.914	1.857	1.848	1.806	8.431
**Model 2 [$(PhS_6)_3Ph$]**	6405.127	797.77	746.85	714.2	708.59	2.067	1.966	1.905	1.895	1.855	8.366

[a]Calculated volume inside a contour of 0.001 electrons/$Bohr^3$ density in optimized structure at B3LYP/6-31G level, [b]Calculated polarizability at B3LYP/6-31G level, [c]Calculated refractive index by Lorentz-Lorenz equation $[(n_\lambda^2 - 1)/(n_\lambda^2 + 2) = (4\pi/3)(\alpha_\lambda/V_{mol})]$, [d]Calculated refractive index at infinite wavelength determined by fitting with the simplified Cauchy's formula ($n_\lambda = n_\infty + D/\lambda^2$).

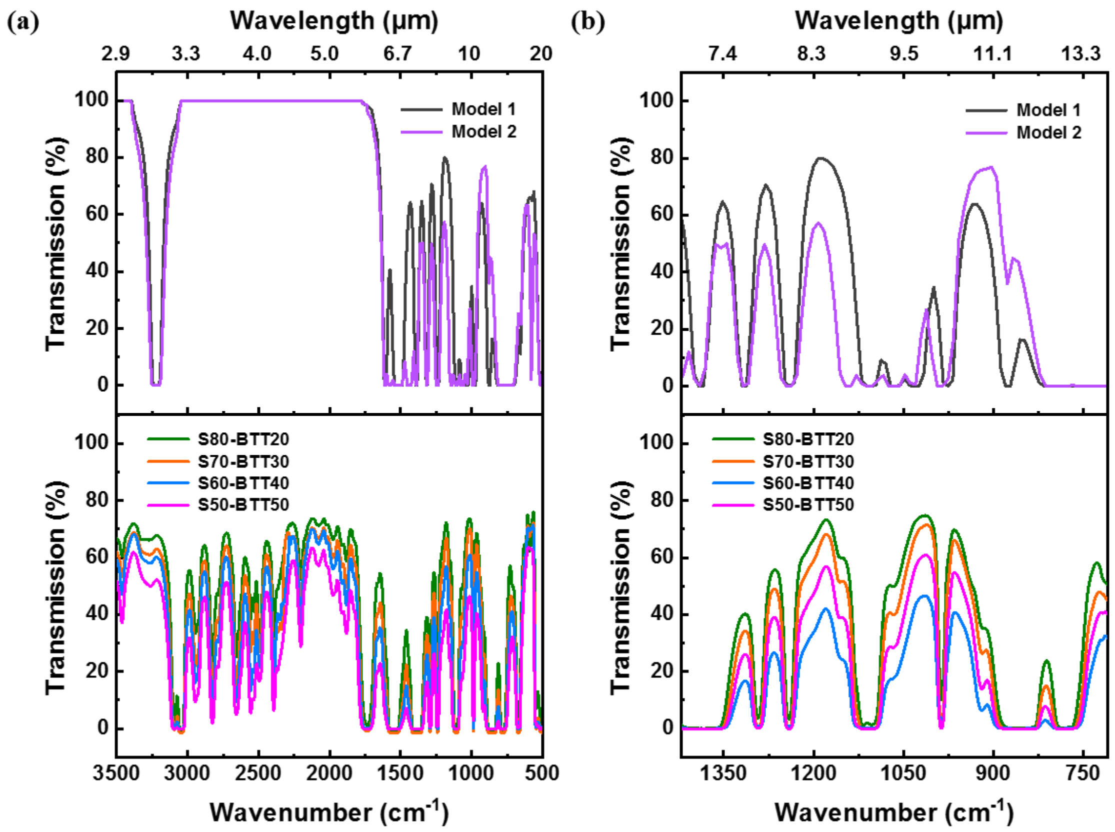

Supplementary figure 20. IR spectra for comparison between experimental and calculated data (a) IR full region and (b) Long-wave infrared (LWIR) region.

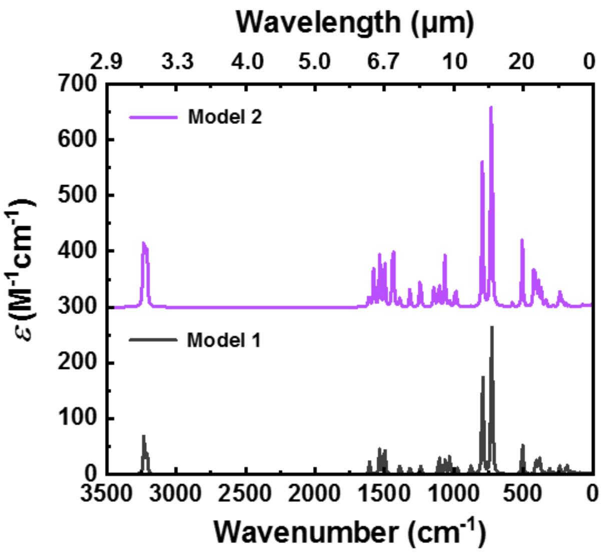

Supplementary figure 21. Calculated IR absorption spectra.

H) DMA curves of poly(S-*r*-BTT) thin films

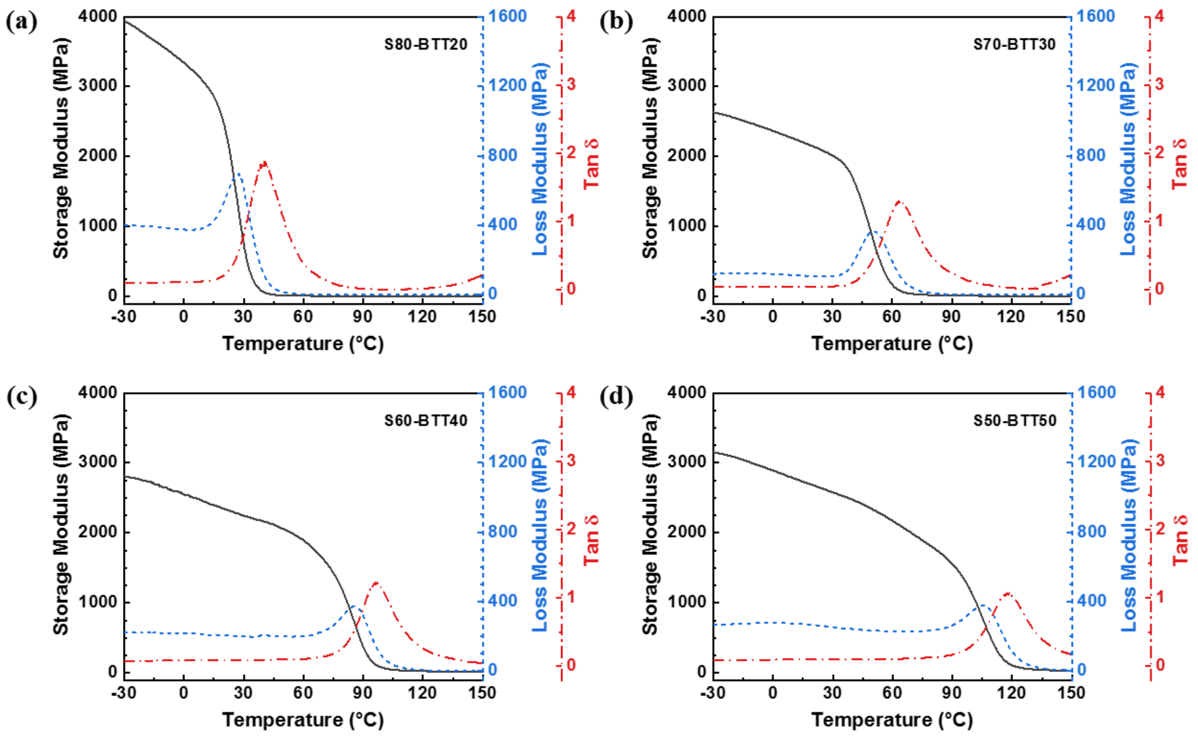

Supplementary figure 22. DMA curves of the poly(S-*r*-BTT) thin film for varying sulfur content (a) S80-BTT20, (b) S70-BTT30, (c) S60-BTT40 and (d) S50-BTT50.

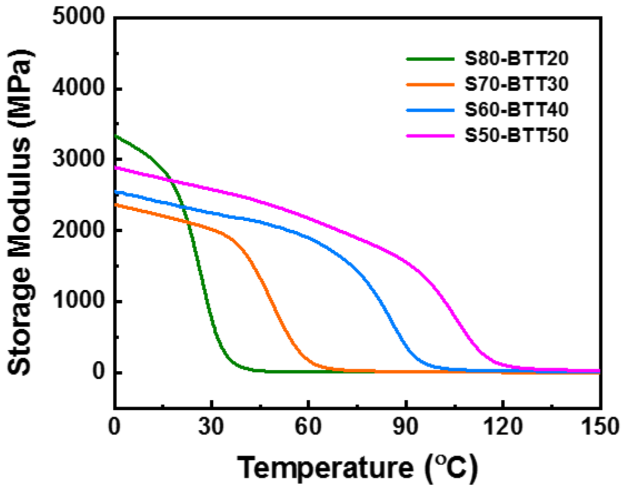

Supplementary figure 23. Plot of storage modulus (E′) for poly(S-*r*-BTT) thin films of varying sulfur content.

Supplementary table 4. Thermal properties of poly(S-*r*-BTT) copolymers of varying sulfur content.

	**$T_{d5\%}$ (°C)[a]**	**$T_{d10\%}$ (°C)[b]**	**T_g (DSC) (°C)**	**T_g (DMA) (°C)**
**S80-BTT20**	205.21	237.80	14.85	40.83
**S70-BTT30**	305.53	345.60	40.39	64.28
**S60-BTT40**	306.94	352.21	66.21	96.31
**S50-BTT50**	369.31	391.66	100.14	118.25

[a]Decomposition temperature, defined as 5 wt% loss, [b]Decomposition temperature, defined as 10 wt% loss.

Supplementary table 5. Cross-linking density of poly(S-*r*-BTT) copolymers of varying sulfur content.

	**S80-BTT20**	**S70-BTT30**	**S60-BTT40**	**S50-BTT50**
ν_e* **(mol/m^3)**	1294	1598	2488	2767

*Cross-linking density calculated as $\nu_e = E'/3RT$, where E′, R, and T are the storage modulus at rubbery plateau regime, ideal gas constant and temperature at E′, respectively.[4] Each E′ values were applied from DMA curves of Supplementary figure 23.

I) Refractive indices of poly(S-*r*-DVB) and poly(S-*r*-BTT) thin films

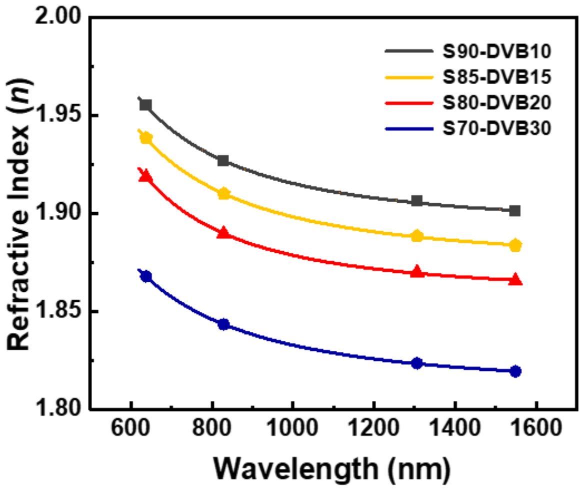

Supplementary figure 24. Average refractive index curves of poly(S-*r*-DVB) films.

Supplementary table 6. Refractive indices of the poly(S-*r*-DVB) films at different wavelengths.

	Wavelength (nm)	n_{TE}[a]	n_{TM}[b]	$n_{av.}$[c]	Δn[d]
**S90-DVB10**	637	1.95502	1.95471	1.95492	0.00031
	829	1.92686	1.92658	1.92677	0.00028
	1306	1.90602	1.90578	1.90594	0.00024
	1549	1.90114	1.90024	1.90084	0.00090
**S85-DVB15**	637	1.93835	1.93816	1.93829	0.00019
	829	1.90991	1.90982	1.90988	0.00009
	1306	1.88835	1.88777	1.88816	0.00058
	1549	1.88362	1.88348	1.88357	0.00014
**S80-DVB20**	637	1.91888	1.91807	1.91861	0.00081
	829	1.89092	1.88673	1.88952	0.00419
	1306	1.87033	1.86857	1.86974	0.00176
	1549	1.86632	1.86391	1.86552	0.00241
**S70-DVB30**	637	1.86818	1.86696	1.86777	0.00122
	829	1.84342	1.84287	1.84324	0.00055
	1306	1.82403	1.82198	1.82335	0.00205
	1549	1.81959	1.81844	1.81921	0.00115

[a]In-plane refractive index, [b]Out-of-plane refractive index, [c]Average refractive index calculated as $n_{av} = [(2n_{TE}^2 + n_{TM}^2)/3]^{1/2}$, [d]In-plane/out-of-plane birefringence calculated as $\Delta n = n_{TE} - n_{TM}$.

Supplementary table 7. Refractive indices of the poly(S-*r*-BTT) films at different wavelengths.

	**Wavelength (nm)**	n_{TE}[a]	n_{TM}[b]	$n_{av.}$[c]	Δn[d]
**S80-BTT20**	637	2.00130	2.00105	2.00122	0.00025
	829	1.96723	1.96710	1.96719	0.00013
	1306	1.94278	1.94254	1.94270	0.00024
	1549	1.93784	1.93564	1.93711	0.00220
**S70-BTT30**	637	1.99078	1.98829	1.98995	0.00249
	829	1.95721	1.95468	1.95637	0.00253
	1306	1.93199	1.93071	1.93156	0.00128
	1549	1.92874	1.92595	1.92781	0.00279
**S60-BTT40**	637	1.96797	1.96686	1.96760	0.00111
	829	1.93816	1.93537	1.93723	0.00279
	1306	1.91345	1.91175	1.91288	0.00170
	1549	1.90919	1.90634	1.90824	0.00285
**S50-BTT50**	637	1.94536	1.94207	1.94426	0.00329
	829	1.91428	1.91231	1.91362	0.00197
	1306	1.89107	1.88900	1.89038	0.00207
	1549	1.88680	1.88591	1.88650	0.00089

[a]In-plane refractive index, [b]Out-of-plane refractive index, [c]Average refractive index calculated as $n_{av} = [(2n_{TE}^2 + n_{TM}^2)/3]^{1/2}$, [d]In-plane/out-of-plane birefringence calculated as $\Delta n = n_{TE} - n_{TM}$.

Supplementary table 8. Optical properties of S70-DIB30, poly(S-*r*-DVB) and poly(S-*r*-BTT) films of varying sulfur content.

	Refractive indices and birefringence at 637 nm				V_{NIR}[e]	n_∞[f]	D[f] [×10^4]
	n_{TE}[a]	n_{TM}[b]	$n_{av.}$[c]	Δn[d]			
**S70-DIB30**	1.86103	1.86100	1.86102	0.00003	19.8	1.8053	2.2459
**S90-DVB10**	1.95502	1.95471	1.95492	0.00031	18.9	1.8899	2.6131
**S85-DVB15**	1.93835	1.93816	1.93829	0.00019	18.1	1.8723	2.6574
**S80-DVB20**	1.91888	1.91807	1.91861	0.00081	18.2	1.8542	2.5732
**S70-DVB30**	1.86818	1.86696	1.86777	0.00122	19.0	1.8093	2.3661
**S80-BTT20**	2.00130	2.00105	2.00122	0.00025	16.5	1.9239	3.1034
**S70-BTT30**	1.99078	1.98829	1.98995	0.00249	16.3	1.9140	3.0436
**S60-BTT40**	1.96797	1.96686	1.96760	0.00111	17.2	1.8959	2.8961
**S50-BTT50**	1.94536	1.94207	1.94426	0.00329	16.8	1.8740	2.8243

[a]In-plane refractive index, [b]Out-of-plane refractive index, [c]Average refractive index calculated as $n_{av} = [(2n_{TE}^2 + n_{TM}^2)/3]^{1/2}$, [d]In-plane/out-of-plane birefringence calculated as $\Delta n = n_{TE} - n_{TM}$. [e]Abbe′s number is given by as $V_{NIR} = (n_{829} - 1)/(n_{637} - n_{1306})$, [f]Calculated refractive index at infinite wavelength determined by fitting with the simplified Cauchy's formula ($n_\lambda = n_\infty + D/\lambda^2$).

J) FT-IR transmission (%) spectra of Ge, commercial polymers, S70-DIB30 1 mm thick windows

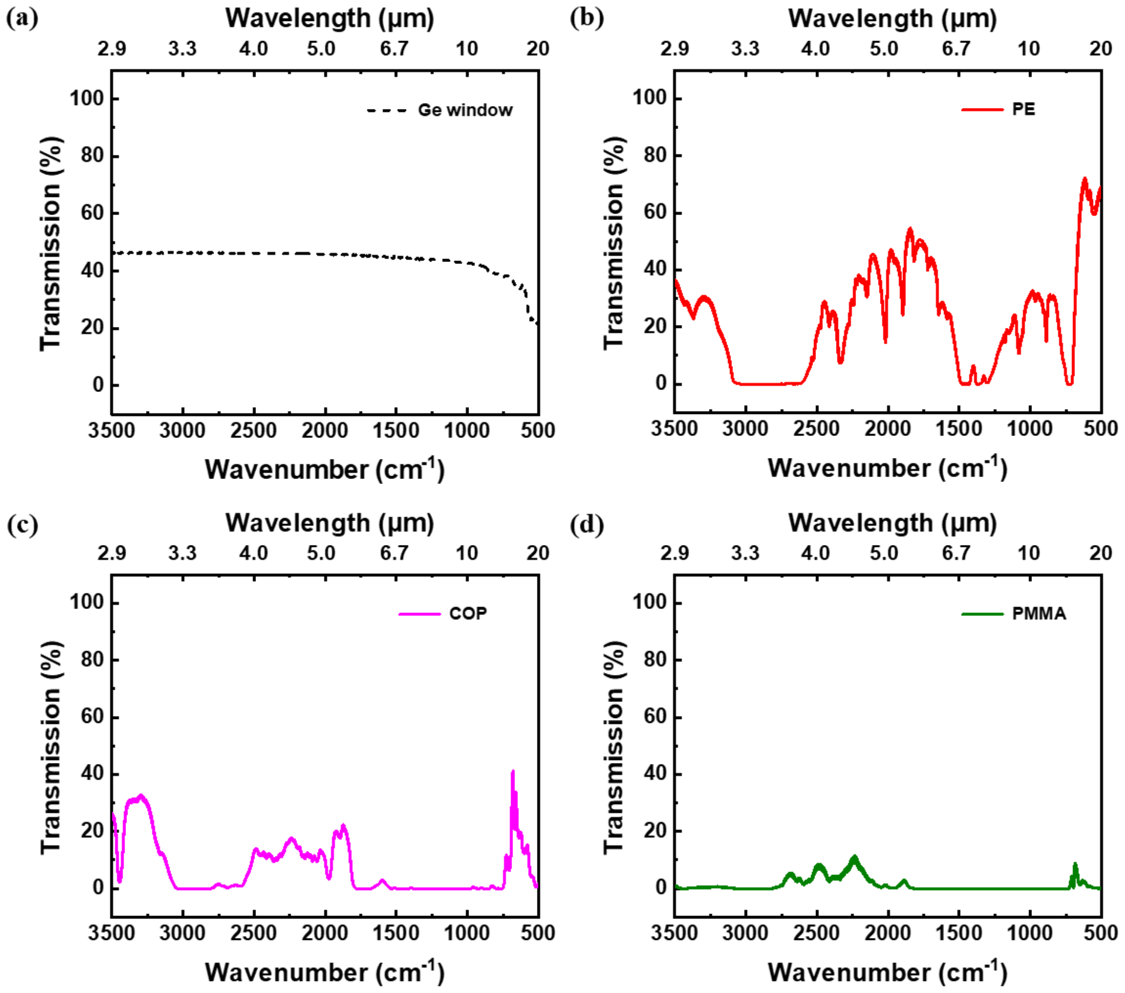

Supplementary figure 25. FT-IR transmission (%) spectra of the Ge and commercial polymers windows (a) Ge window, (b) PE (polyethylene), (c) COP (cyclic olefin copolymer) and (d) PMMA (poly(methyl methacrylate)) (commercial polymer windows were measured 5 times).

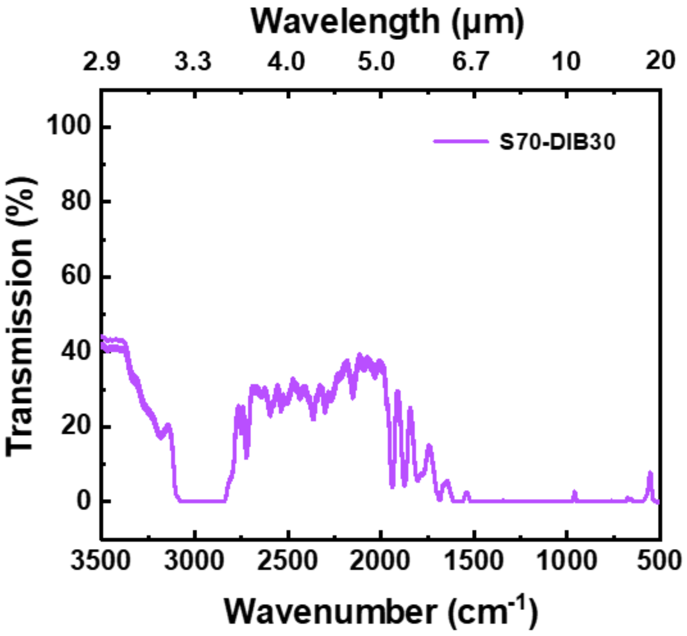

Supplementary figure 26. FT-IR transmission (%) spectra of the S70-DIB30 window (5 measurements).

K) FT-IR transmission (%) spectra of S-DVB and S-BTT 1 mm thick windows

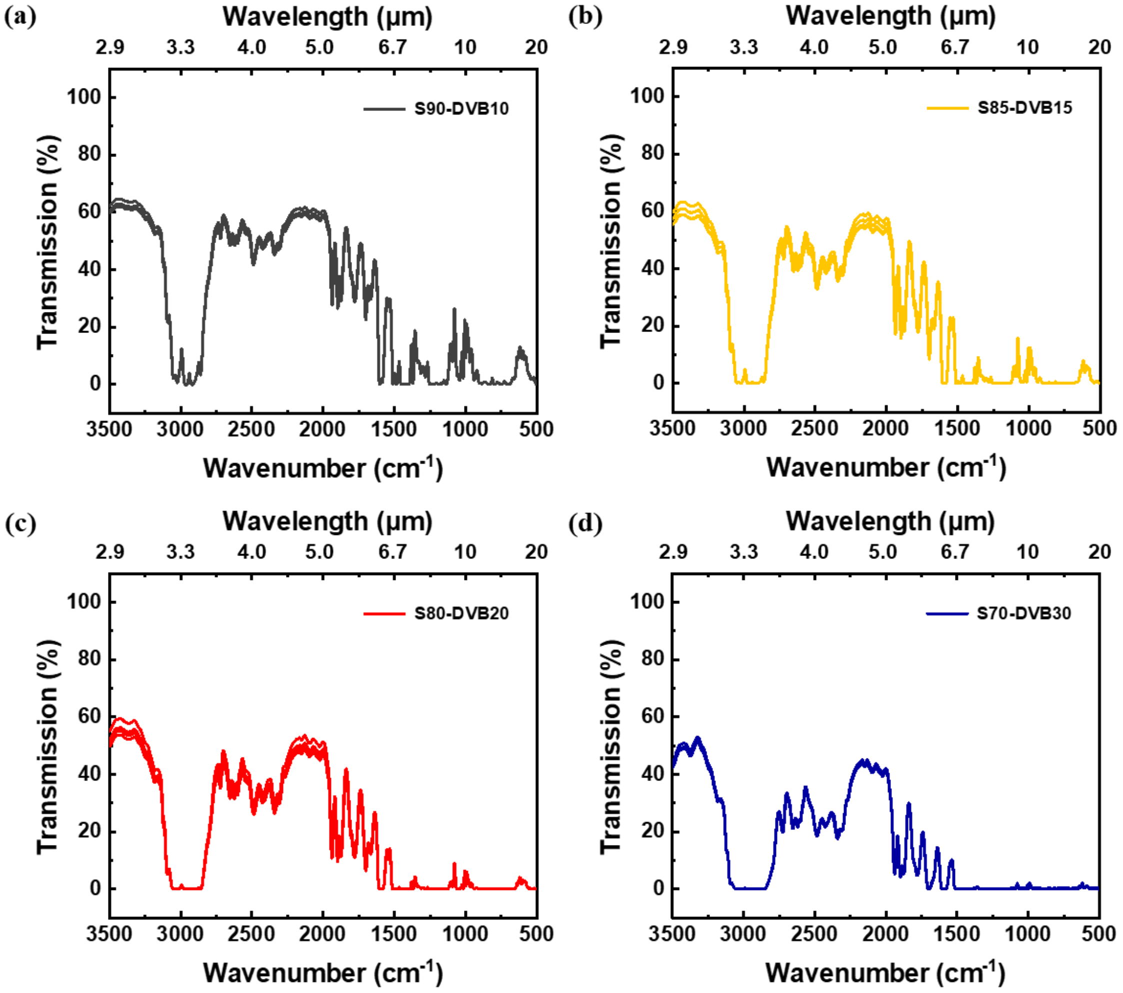

Supplementary figure 27. FT-IR transmission (%) spectra of the poly(S-*r*-DVB) windows (5 measurements) (a) S90-DVB10, (b) S85-DVB15, (c) S80-DVB20 and (d) S70-DVB30.

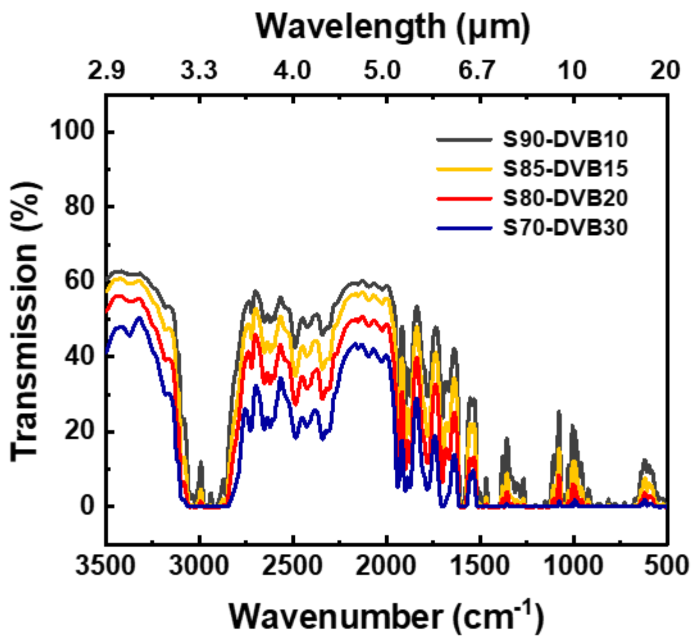

Supplementary figure 28. Average FT-IR transmission (%) spectra of poly(S-*r*-DVB) windows.

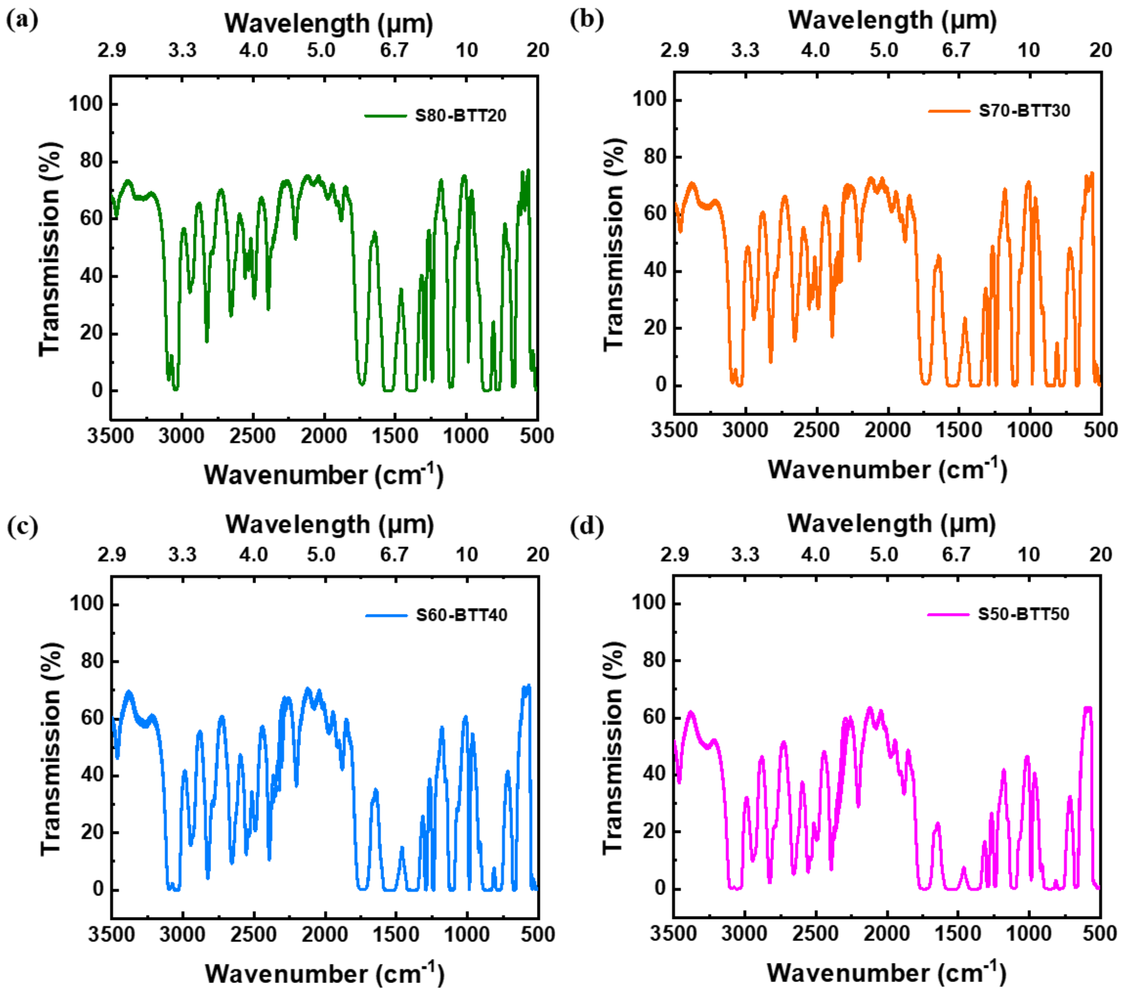

Supplementary figure 29. FT-IR transmission (%) spectra of the poly(S-*r*-BTT) windows (5 measurements) (a) S80-BTT20, (b) S70-BTT30, (c) S60-BTT40 and (d) S50-BTT50.

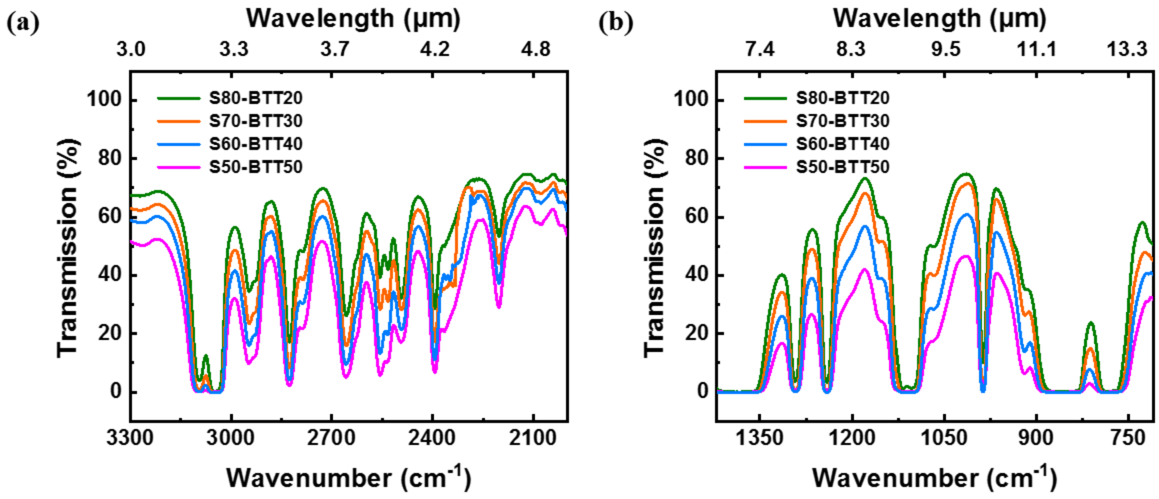

Supplementary figure 30. Average FT-IR transmission (%) spectra of poly(S-*r*-BTT) windows (a) MWIR region and (b) LWIR region.

L) Average FT-IR transmission (%) spectrum of Ge and polymer windows

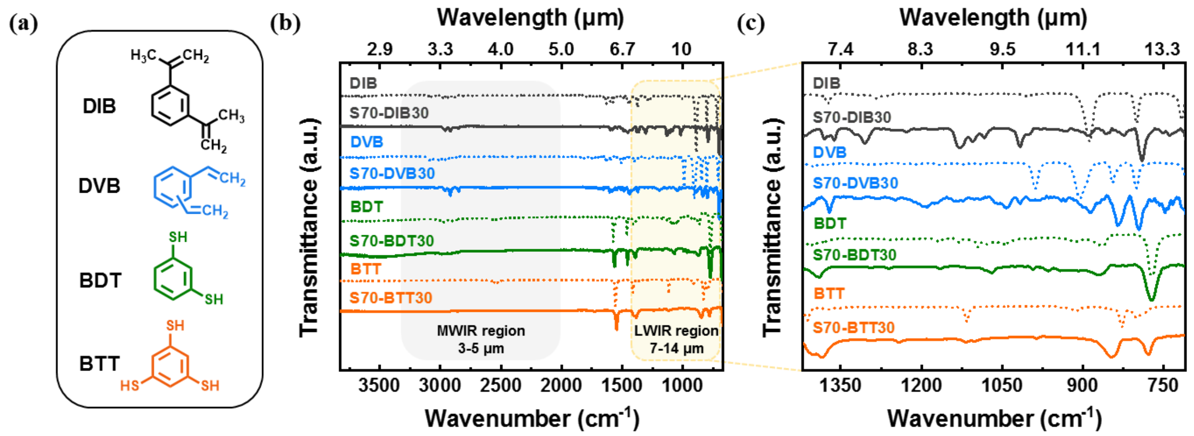

Supplementary figure 31. (a) Comonomer structure used for inverse vulcanization: DIB (1,3-diisopropenylbenzene), DVB (Divinylbenzene) BDT (1,3-benzenedithiol) and BTT (1,3,5-benzenetrithiol), (b) Expended FT-IR spectra of monomers and polymers and (c) LWIR spectra of monomers and polymers.

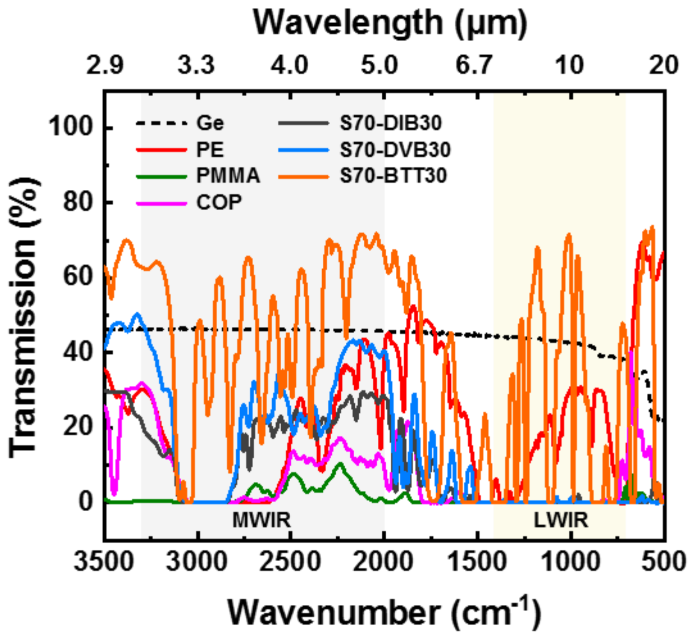

Supplementary figure 32. Average FT-IR transmission (%) spectra of the Ge and polymer windows.

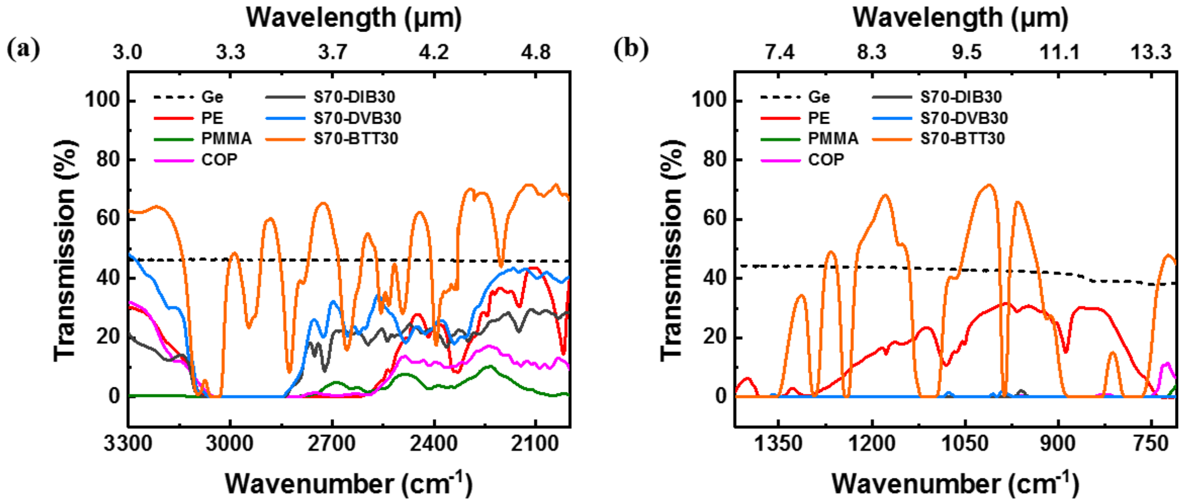

Supplementary figure 33. Average FT-IR transmission (%) spectra of the Ge and polymer windows (a) MWIR region and (b) LWIR region.

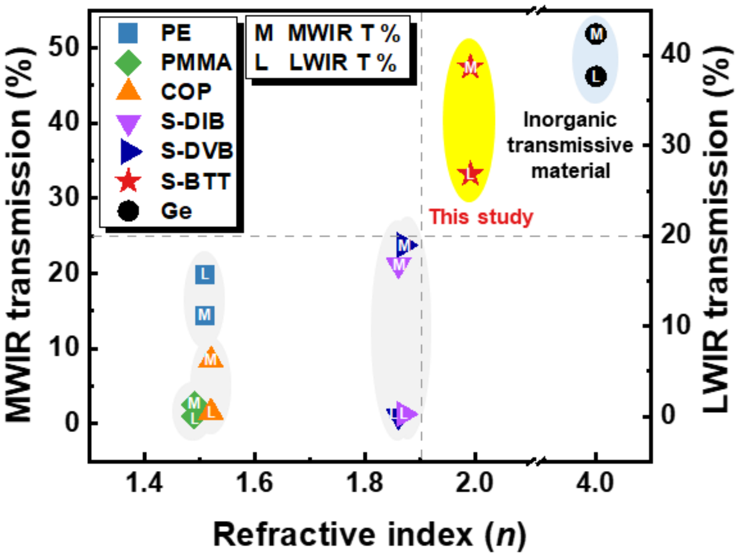

Supplementary figure 34. Optical properties of Ge and polymer windows (S-DIB, S-DVB and S-BTT with sulfur content of 70 wt%). MWIR transmission (%) indicate the average transmission in the 3-5 μm (3300-2000 cm^{-1}) region and LWIR transmission (%) indicate the average transmission in the 7–14 μm,(1420-710 cm^{-1}).

Supplementary table 9. Comparison of optical properties with Ge and polymer windows

	Sulfur content (wt%)	Thickness (mm)	Refractive index (*n*)[a]	Transmission (%)		Ref
				MWIR	LWIR	
**Ge (Germanium)**	-	1.087	4.00[b] * approximate value in the 2-14 μm region	46.19	42.37	-
**S-BTT**	70	1.139	1.99	47.44	26.83	-
**S-DVB**	70	1.276	1.87	23.77	0.24	5
**S-DIB**	70	1.281	1.86	21.16	0.08	6
**PE (Polyethylene)**	-	1.090	1.51	14.32	15.68	-
**COP (Cyclic olefin copolymer)**	-	1.098	1.52[c]	8.45	0.45	-
**PMMA (Poly(methyl methacrylate))**	-	1.153	1.49	2.53	0.05	-

[a]Measured at 637 nm, [b]Edmund Optics and [c]JSR Corporation.

M)FT-IR transmission (%) spectra of S70-BTT30 windows according to hot-pressing condition

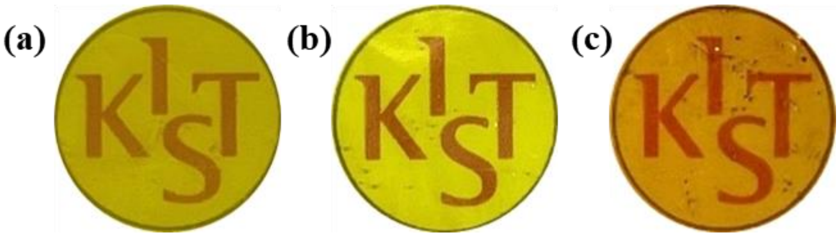

Supplementary figure 35. Photographs of S70-BTT30 windows according to hot-processing temperature (thickness ca. 1 mm) (a) 150 °C, (b) 185 °C and (c) 230 °C (processing time: 30 min).

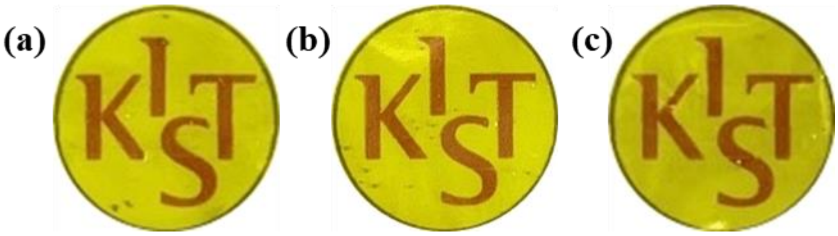

Supplementary figure 36. Photographs of S70-BTT30 windows according to hot-processing time (thickness ca. 1 mm) (a) 10 min, (b) 30 min and (c) 1 hr (processing temperature : 185 °C).

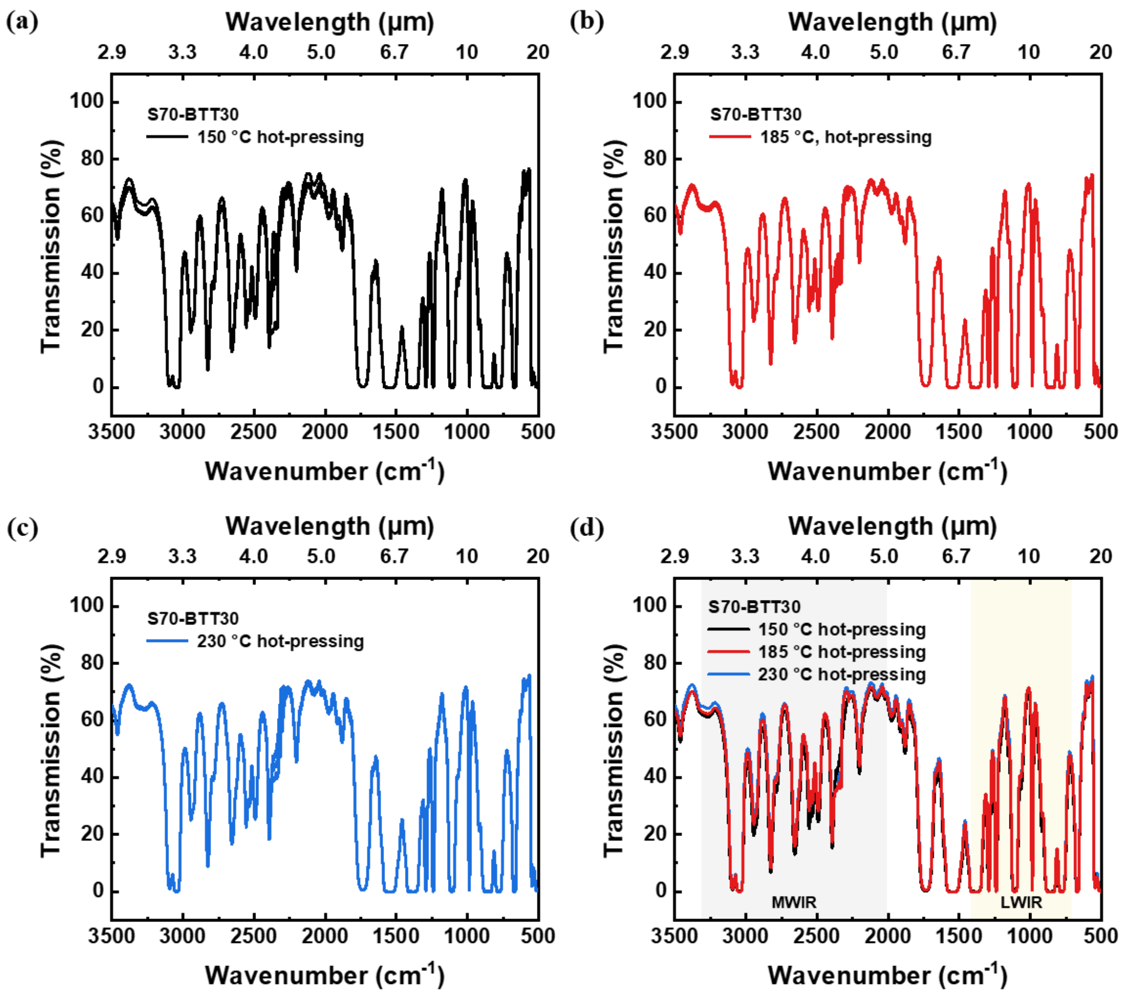

Supplementary figure 37. FT-IR transmission (%) spectra of the S70-BTT30 windows according to hot-pressing temperature (5 measurements) (a) 150 °C (thickness: 1.274 mm), (b) 185 °C (thickness: 1.139 mm), (c) 230 °C (thickness: 1.127 mm) and (d) average FT-IR transmission (%) spectra.

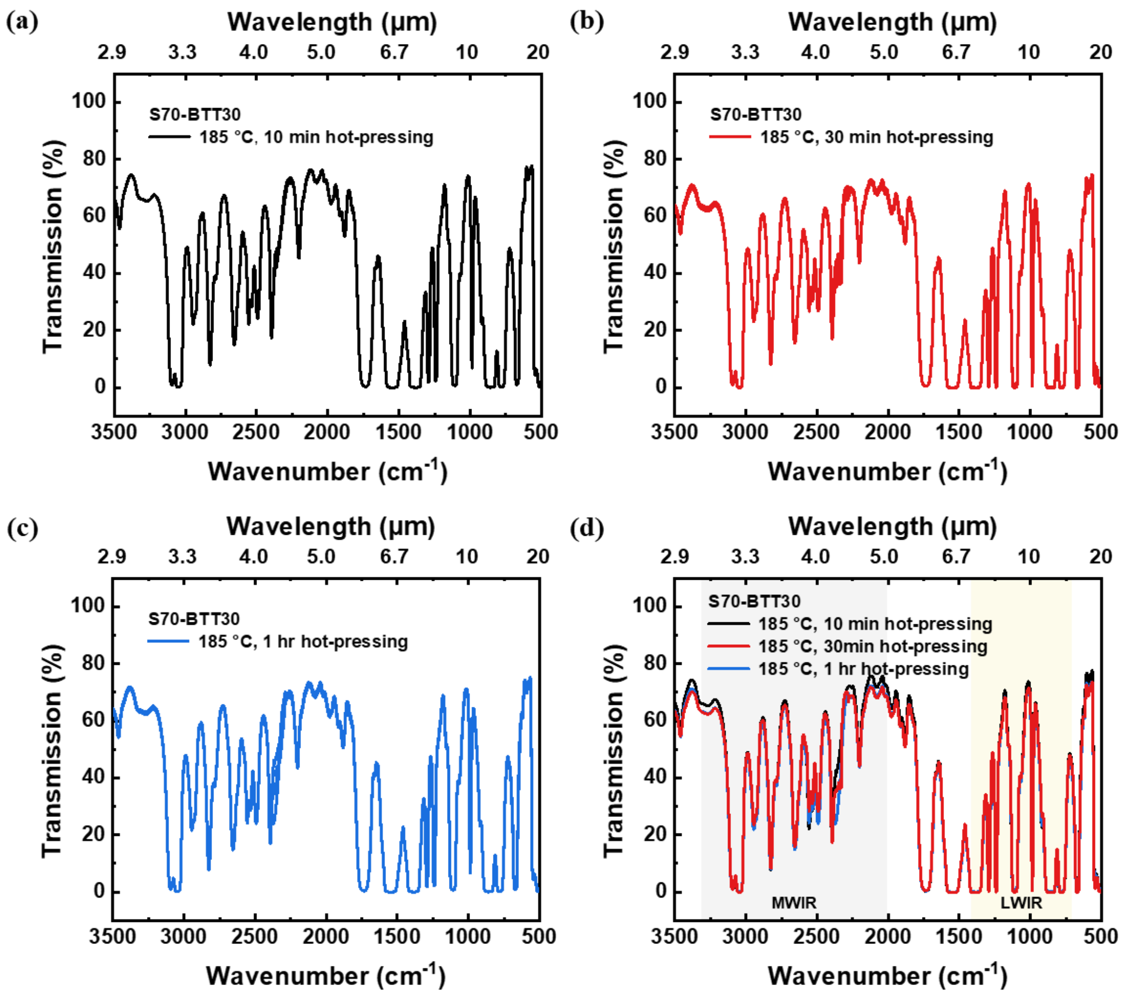

Supplementary figure 38. FT-IR transmission (%) spectra of the S70-BTT30 windows according to hot-pressing time at 185°C (5 measurements) (a) 10 min (thickness: 1.212 mm), (b) 30 min (thickness: 1.139 mm), (c) 1 hr (thickness: 1.191 mm) and (d) average FT-IR transmission (%) spectra.

N) Optical stability of S70-DIB30, S70-DVB30 and S70-BTT30 windows

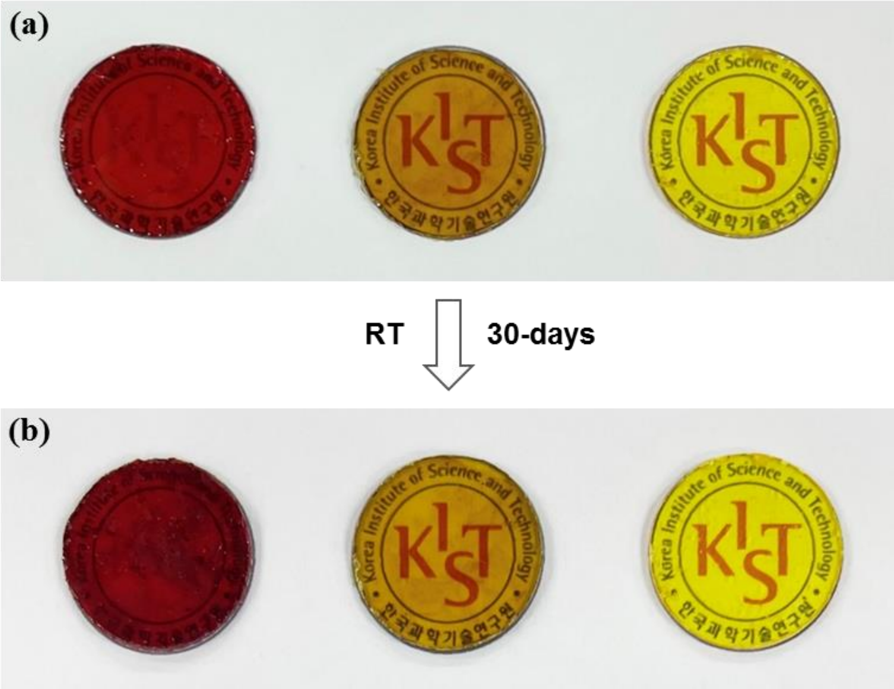

Supplementary figure 39. Photographs of S70-DIB30, S70-DVB30 and S70-BTT30 windows (thickness ca. 1 mm) (a) initial and (b) stored at room temperature for 30-days (from left to right).

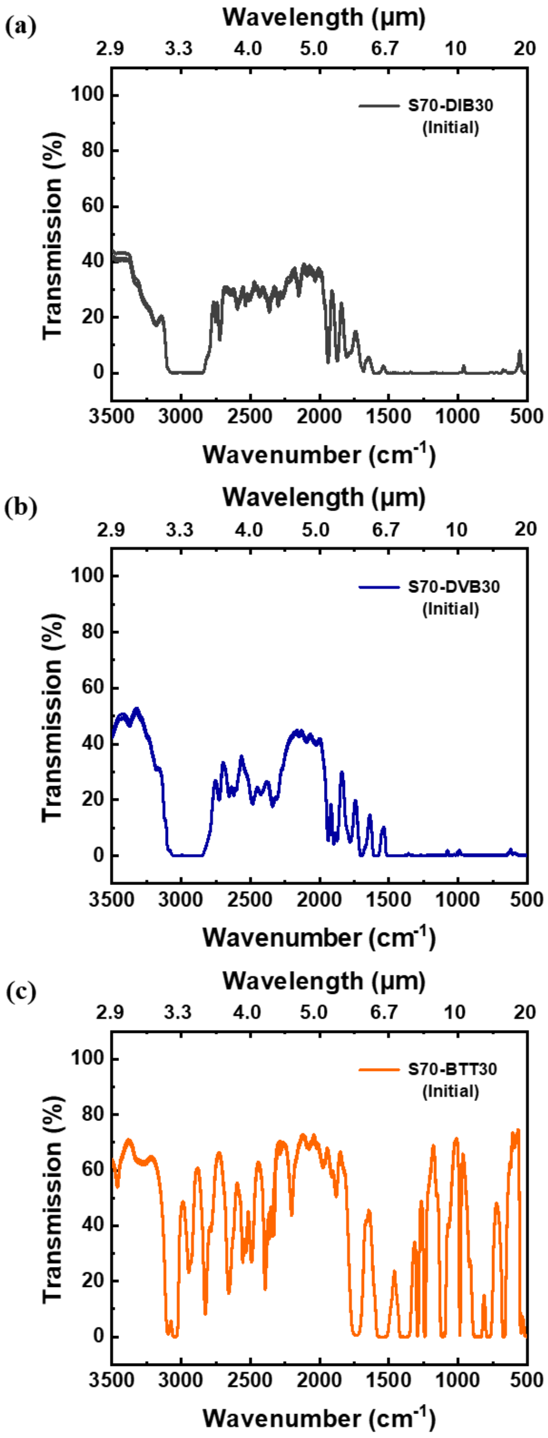

Supplementary figure 40. FT-IR transmission (%) spectra of the initial polymer windows (5 measurements) (a) S70-DIB30 (thickness: 1.281 mm), (b) S70-DVB30 (thickness: 1.276 mm) and (c) S70-BTT30 (thickness: 1.139 mm).

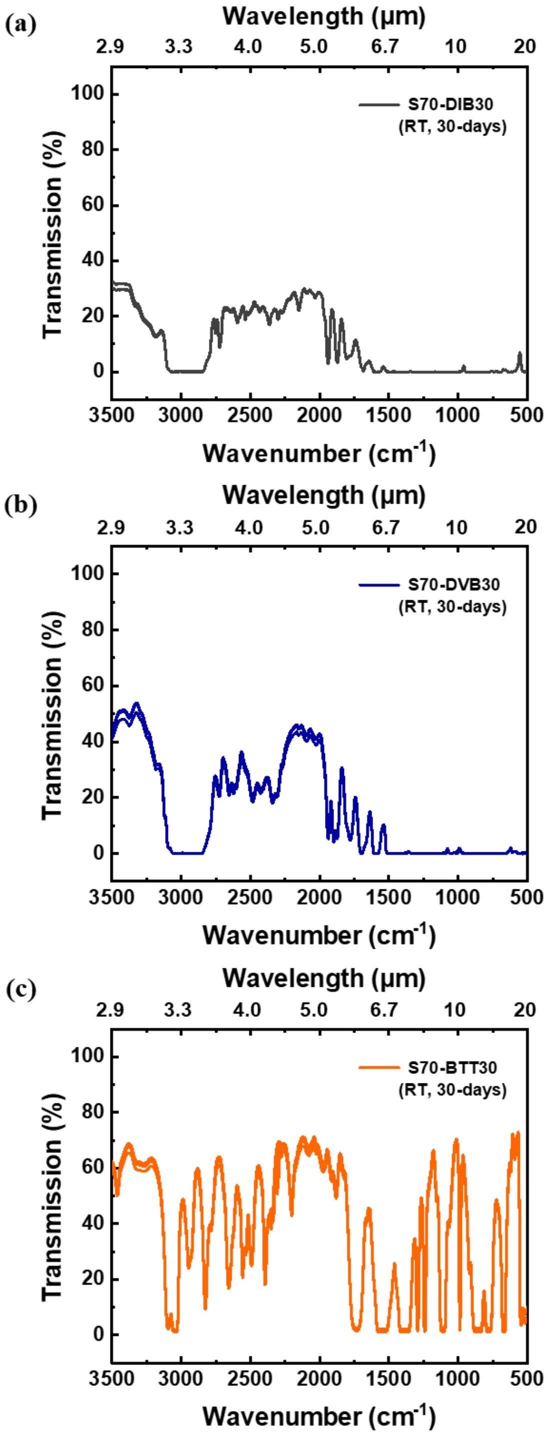

Supplementary figure 41. FT-IR transmission (%) spectra of the polymer windows stored at room temperature for 30-days (5 measurements) (a) S70-DIB30 (thickness: 1.281 mm), (b) S70-DVB30 (thickness: 1.276 mm) and (c) S70-BTT30 (thickness: 1.139 mm).

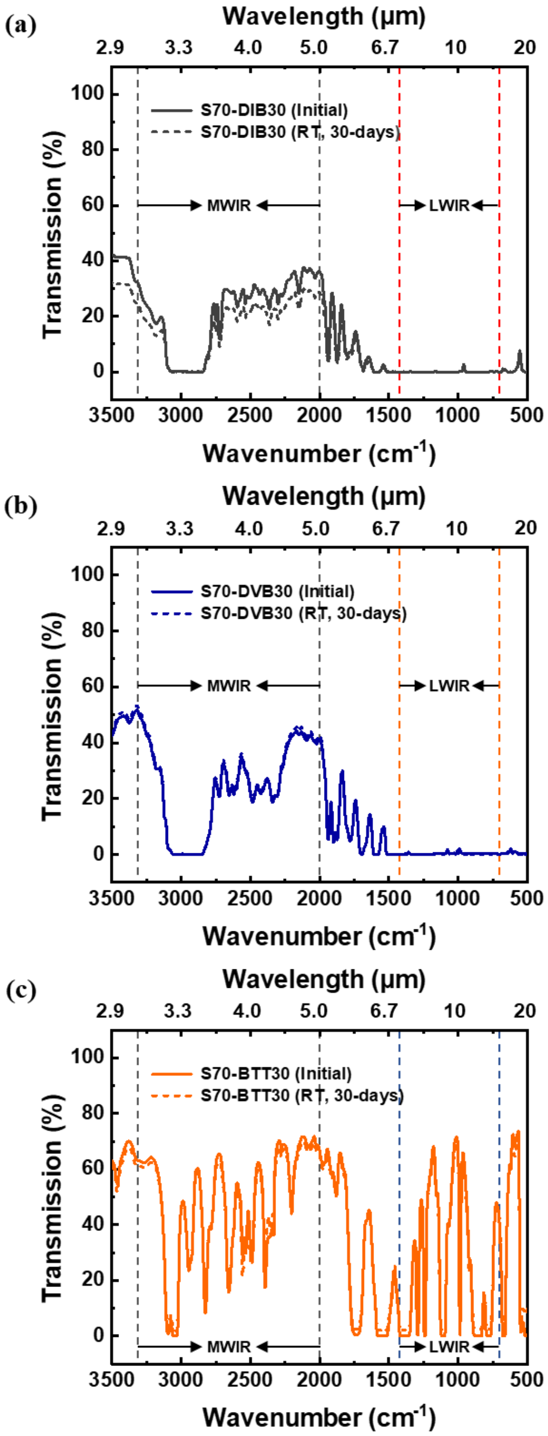

Supplementary figure 42. Average FT-IR transmission (%) spectra of polymer windows, initial and stored at room temperature for 30-days (a) S70-DIB30 (thickness: 1.281 mm), (b) S70-DVB30 (thickness: 1.276 mm) and (c) S70-BTT30 (thickness: 1.139 mm).

O) Elemental analyzes of S70-DIB30, poly(S-*r*-DVB) and poly(S-*r*-BTT) copolymers

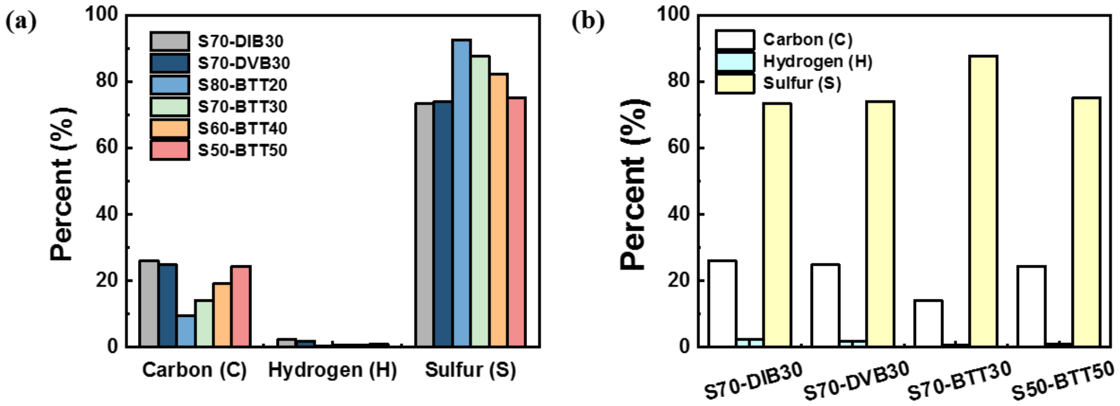

Supplementary figure 43. Elemental analysis of (a) S70-DIB30, S70-DVB30 and poly(S-*r*-BTT) copolymers for varying sulfur content, (b) S70-DIB30, S70-DVB30, S70-BTT30 and S50-BTT50.

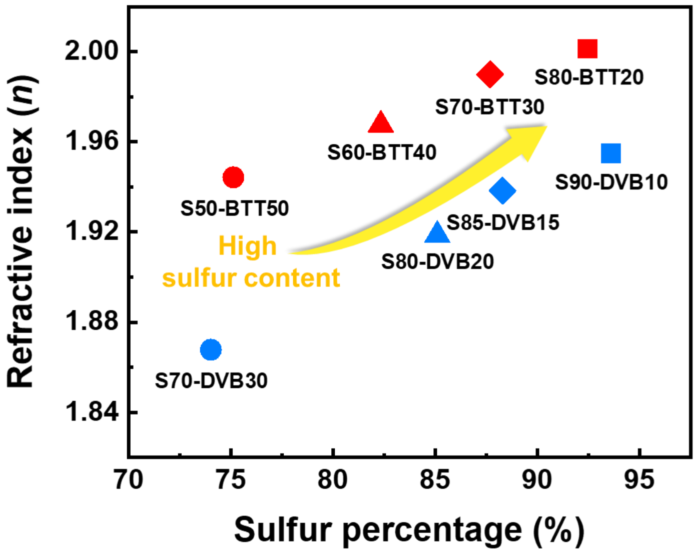

Supplementary figure 44. Refractive indices by sulfur percent (%) of the poly(S-*r*-DVB) and poly(S-*r*-BTT) films.

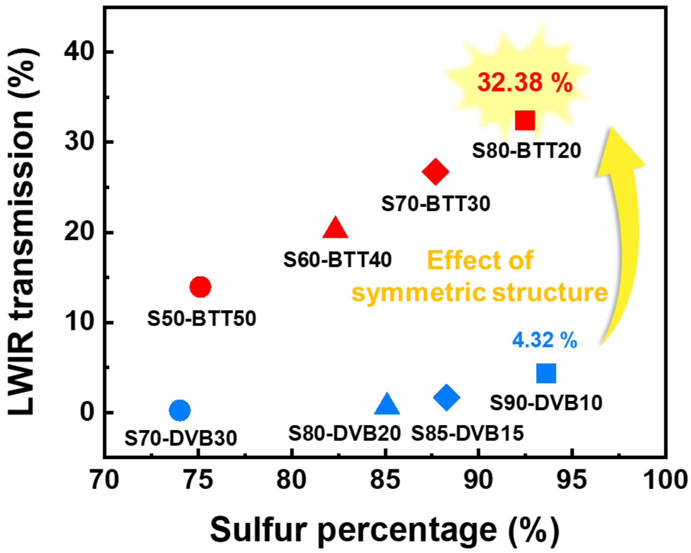

Supplementary figure 45. LWIR transmission (%) by sulfur percent (%) of the poly(S-*r*-DVB) and poly(S-*r*-BTT) windows (thickness ca. 1 mm).

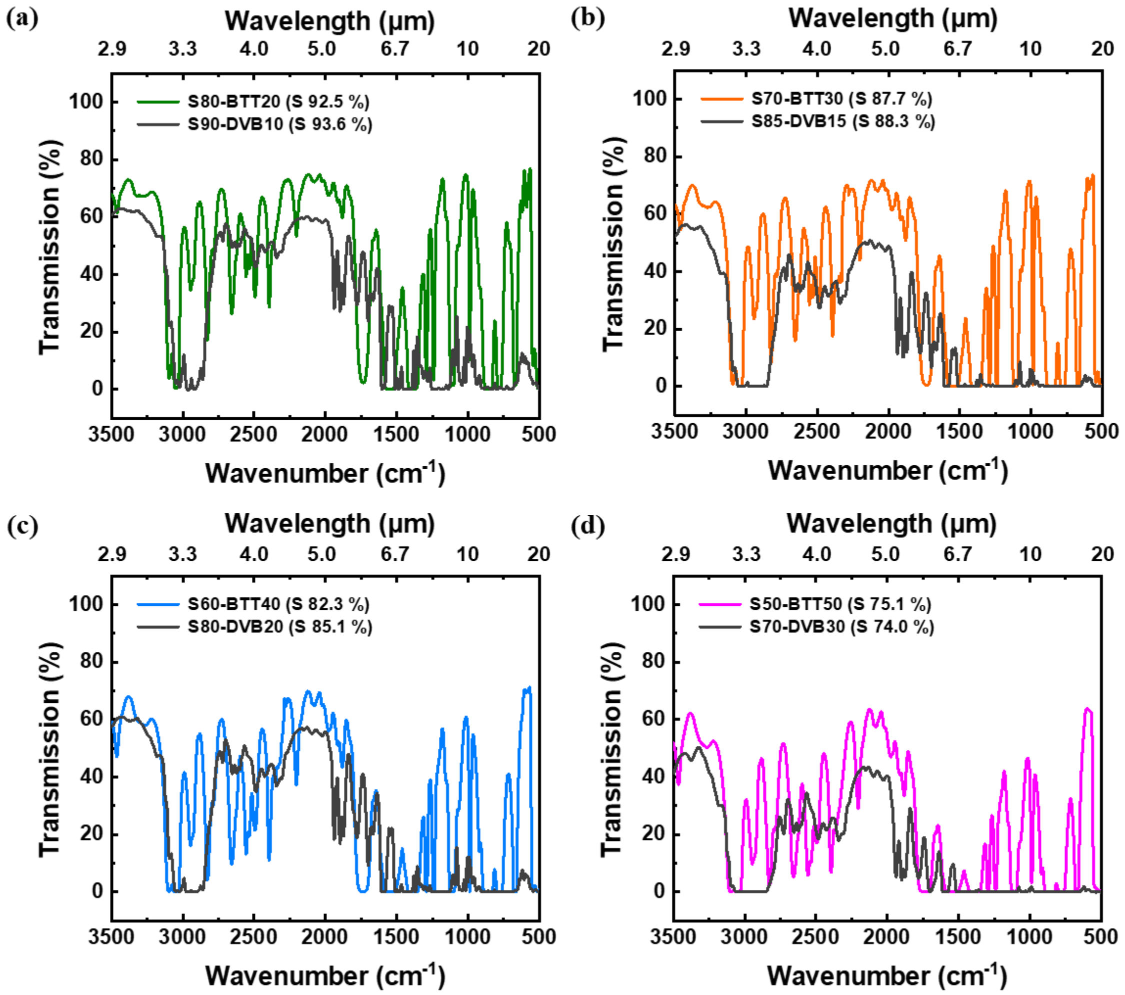

Supplementary figure 46. FT-IR transmission (%) spectra of poly(S-*r*-DVB) and poly(S-*r*-BTT) windows with same percentage of sulfur (thickness ca. 1 mm) (a) S80-BTT20&S90-DVB10, (b) S70-BTT30&S85-DVB15, (c) S60-BTT40&S80-DVB20 and (d) S50-BTT50&S70-DVB30.

Supplementary table 10. Elemental analysis of the S70-DIB30, poly(S-*r*-DVB) and poly(S-*r*-BTT) copolymers for varying sulfur content.

	Content (wt%)		Carbon (C) %	Hydrogen (H) %	Nitrogen (N) %	Sulfur (S) %	%
	Sulfur	Comonomer					
**S70-DIB30**	70	30	26.072	2.228	0.015	73.299	101.614
**S90-DVB10**	90	10	7.930	0.636	0.000	93.620	102.186
**S85-DVB15**	85	15	12.394	0.993	0.000	88.292	101.679
**S80-DVB20**	80	20	15.735	1.289	0.000	85.100	102.124
**S70-DVB30**	70	30	24.754	1.881	0.006	74.018	100.659
**S80-BTT20**	80	20	9.500	0.321	0.015	92.473	102.309
**S70-BTT30**	70	30	14.009	0.483	0.015	87.682	102.189
**S60-BTT40**	60	40	19.118	0.683	0.000	82.337	102.138
**S50-BTT50**	50	50	24.339	0.896	0.000	75.120	100.355

Supplementary table 11. Elemental analysis of the S70-DIB30, S70-DVB30, S70-BTT30 and S50-BTT50.

	Content (wt%)		Carbon (C) %	Hydrogen (H) %	Sulfur (S) %	S/C ratio
	Sulfur	Comonomer				
**S70-DIB30**			26.072	2.228	73.299	2.811
**S70-DVB30**	70	30	24.754	1.881	74.018	2.990
**S70-BTT30**			14.009	0.483	87.682	6.259
**S50-BTT50**	50	50	24.339	0.896	75.120	3.086

P) Infrared (IR) imaging experiments

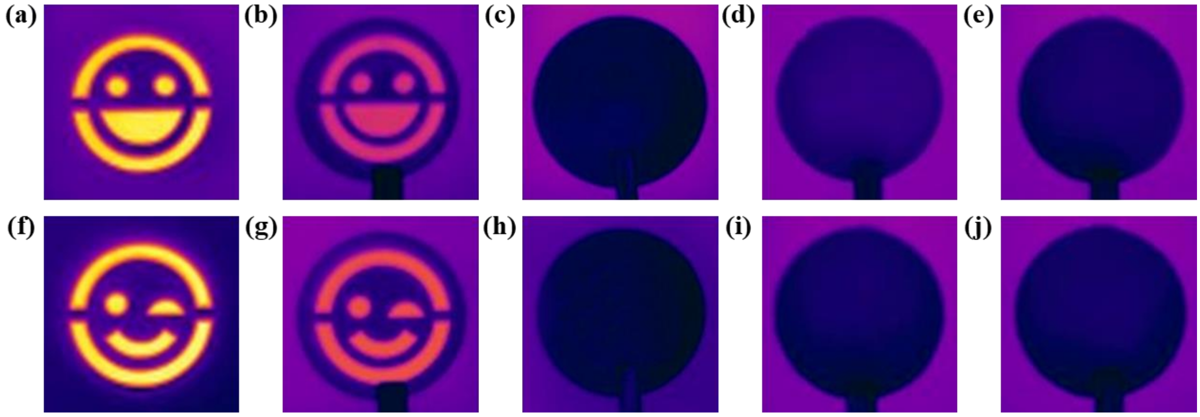

Supplementary figure 47. LWIR region images of various patterned PMMA mask through Ge and various polymer windows (thickness ca. 1 mm) (a)&(f) direct image (without windows), (b)&(g) Ge, (c)&(h) PMMA (Poly(methyl methacrylate), (d)&(i) S70-DIB30 and (e)&(j) S70-DVB30.

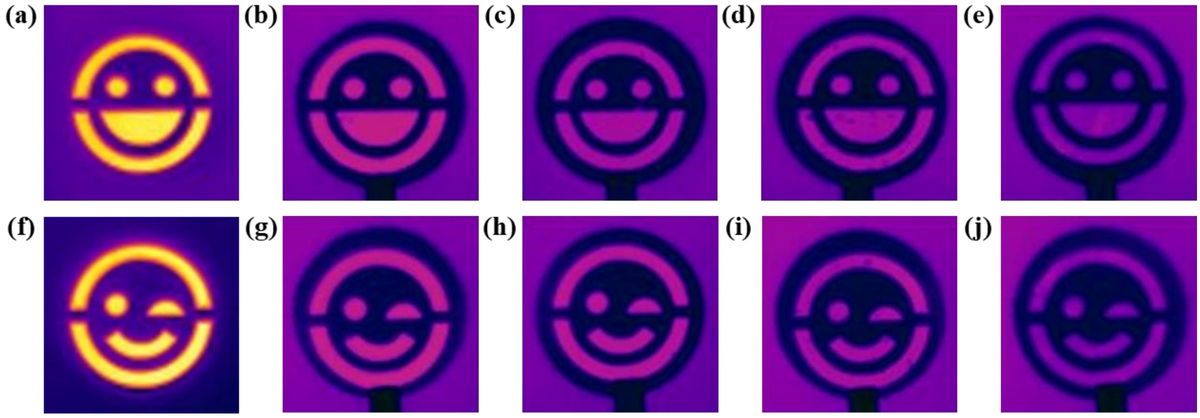

Supplementary figure 48. LWIR region images of various patterned PMMA mask through poly(S-*r*-BTT) windows (thickness ca. 1 mm) (a)&(f) direct image (without windows), (b)&(g) S80-BTT20, (c)&(h) S70-BTT30, (d)&(i) S60-BTT40 and (e)&(j) S50-BTT50.

III) Supplementary References

1. Tristan S. Kleine. et al. Infrared Fingerprint Engineering: A Molecular-Design Approach to Long-Wave Infrared Transparency with Polymeric Materials. *Angew. Chem. Int. Ed*. **58**, 17656-17660 (2019).

2. Chung, W. J. et al. The use of elemental sulfur as an alternative feedstock for polymeric materials. *Nat. Chem*. **5**, 518-524 (2013).

3. Han Y and Elliott J. Molecular dynamics simulations of the elastic properties of polymer/carbon nanotube composites. *Comput Mater Sci*. **39**, 315-323 (2007).

4. P. J. Flory, Molecular theory of rubber elasticity, *Polymer*, **20**, 1317-1320 (1979).

5. Sangwoo Park. et al. Inverse Vulcanization Polymers with Enhanced Thermal Properties via Divinylbenzene Homopolymerization-Assisted Cross-Linking. *ACS Macro Lett*. **8**, 1670-1678 (2019).

6. Jared J. et al. New Infrared Transmitting Material via Inverse Vulcanization of Elemental Sulfur to Prepare High Refractive Index Polymers. *Adv. Mater*. **26**, 3014-3018 (2014).
