# Supplementary material for: Long-wave infrared transparent sulfur polymers enabled by symmetric thiol cross-linker
Source: Nat Commun. 2023 May 19;14:2866. doi: 10.1038/s41467-023-38398-5 (PMC10199000; doi:10.1038/s41467-023-38398-5)
Supplement: Supplementary file 3 — Description of Additional Supplementary Files [file 41467_2023_38398_MOESM3_ESM.pdf]

Description of Additional Supplementary Files

File Name: Supplementary Movie 1
Description: **LWIR imaging experiment**
LWIR imaging of 1 mm thick PMMA, S70-BTT30, S70-DIB30 and S70-DVB30 windows (clockwise from left upper). The S70-DIB30, S70-DVB30 and PMMA windows clearly showed opaque properties in the LWIR region, while the S-BTT windows showed excellent transparency in the LWIR region.
